# Potential structural trait markers of depression in the form of alterations in the structures of subcortical nuclei and structural covariance network properties

**DOI:** 10.1016/j.nicl.2021.102871

**Published:** 2021-11-03

**Authors:** Ge Xiong, Daifeng Dong, Chang Cheng, Yali Jiang, Xiaoqiang Sun, Jiayue He, Chuting Li, Yidian Gao, Xue Zhong, Haofei Zhao, Xiang Wang, Shuqiao Yao

**Affiliations:** a Medical Psychological Center, The Second Xiangya Hospital, Central South University, Changsha, Hunan 410011, China; b Medical Psychological Institute of Central South University, Changsha, Hunan 410011, China; c China National Clinical Research Center on Mental Disorders (Xiangya), Changsha, Hunan 410011, China; d School of Psychology, South China Normal University, Guangzhou 510631, China

**Keywords:** Major depressive disorder, Remitted depression, Subcortical volume, Structural covariance network, State-independent alteration

## Abstract

It has been proposed recently that major depressive disorder (MDD) could represent an adaptation to conserve energy after the perceived loss of an investment in a vital source, such as group identity, personal assets, or relationships. Energy conserving behaviors associated with MDD may form a persistent marker in brain regions and networks involved in cognition and emotion regulation. In this study, we examined whether subcortical regions and volume-based structural covariance networks (SCNs) have state-independent alterations (trait markers).

First-episode drug-naïve currently depressed (cMDD) patients (N = 131), remitted MDD (RD) patients (N = 67), and healthy controls (HCs, N = 235) underwent structural magnetic resonance imaging (MRI). Subcortical gray matter volumes (GMVs) were calculated in FreeSurfer software, and group differences in GMVs and SCN were analyzed.

Compared to HCs, major findings were decreased GMVs of left pallidum and pulvinar anterior of thalamus in the cMDD and RD groups, indicative of a trait marker. Relative to HCs, subcortical SCNs of both cMDD and RD patients were found to have reduced small-world-ness and path length, which together may represent a trait-like topological feature of depression.

In sum, the left pallidum, left pulvinar anterior of thalamus volumetric alterations may represent trait marker and reduced small-world-ness, path length may represent trait-like topological feature of MDD.

## Highlights

• Reduced GMV in the left pallidum and PuA of thalamus were trait-like in MDD.
• Reduced GMV in the right pallidum and VL of thalamus were state-dependent.
• Altered topological properties of subcortical SCNs show trait-like features.

## 1 Introduction

Major depressive disorder (MDD) is a psychiatric disorder with high recurrence (Eaton et al., 2008, Steinert et al., 2014). For many years, researchers have attempted to construct a conceptual framework that explains how brain characteristics are related to MDD-associated behavioral alterations and depressive symptoms (Disner et al., 2011, Drevets et al., 2008, Mayberg, 2003, Phillips et al., 2003). The notion that depressive predispositions (e.g. depressogenic beliefs, clinical features of depression, triggered biological reactions) underlain by brain area/network characteristics may be an anachronistic manifestation of an evolutionarily based program—that is, a set of traits that were once-adaptive but are maladaptive in modern society—has gained interest (Beck and Bredemeier, 2016). According to this view, genetic vulnerabilities and experiential risk factors contribute to maladaptive progression and lead to the development of depressogenic beliefs (Beck and Bredemeier, 2016), which, in turn, exacerbate negative processing biases and stress reactivity. In individuals with depressive predispositions, a depressive episode may produce residua in brain structures (Schmaal and van Velzen, 2019). This effect in individuals who have MDD may be evident as a stable trait marker that can be observed across depressive stages. Such a persistent marker may represent core depressive factors in brain structure and may contribute to the maintenance of depressive symptoms and ongoing risk of MDD episode recurrence. However, there are not yet sufficient data to understand the involvement of aforementioned neural mechanisms across depressive stages.

Previously, researchers have related structural alterations in corticolimbic regions in the brains of patients with MDD to reduced interactions that would result in impaired mood regulation and reduced cognitive control, and they have suggested that these alterations may represent stable characteristics of MDD that influence bottom-up and top-down neural pathways (Disner et al., 2011, Mayberg, 1997, Phillips et al., 2003). Consistent with this perspective of depression, empirical studies of structural magnetic resonance imaging (MRI) have confirmed state-independent alterations (consistent with a trait marker) of cortical thickness and gyrification in corticolimbic structures (e.g. frontal cortex, caudal anterior cingulate cortex, posterior cingulate cortex, and temporal pole) of both first-episode drug-naïve currently-depressed MDD (cMDD) patients and remitted MDD (RD) patients (van Eijndhoven et al., 2013, Xiong et al., 2019).

Although several studies have explored cortical trait markers, little study has been reported to potential trait markers of subcortical structure. Empirical studies found altered amygdalar (van Eijndhoven et al., 2009) and hippocampal (Arnone et al., 2012) gray matter volumes (GMVs) as potential state markers. Previous studies seem to only focus on whether the GMVs alterations of the hippocampus and amygdala are the trait or state markers of MDD without considering other subcortical structures. In the cognitive neurobiological model of MDD (Disner et al., 2011), the amygdala, thalamus and hippocampus play an important role in the processing of bottom-up pathway. Negative stimuli, including stress-inducing stimuli, are routed by the thalamus through a cognitive hierarchy in which cortical regions are the highest level [e.g., dorsolateral prefrontal cortex (DLPFC), ventrolateral prefrontal cortex (VLPFC) and medial prefrontal cortex (MPFC)]. This routing is thought to begin with the amygdala whose activation shows a bias for negative stimuli. The information about negative stimuli is to be sent to a subcortical circuit, including the hippocampus, for additional processing, and then finally to a cortical circuit where self-referential schemas and rumination are induced, thus promoting depressive symptoms. Furthermore, a group of subcortical nuclei (the main components of the basal ganglia), including the caudate, putamen, NAc, and pallidum have been reported to play a critical role in core physiological depressive symptoms, such as psychomotor symptoms (Buyukdura et al., 2011) and anhedonia (Berridge and Kringelbach, 2015, Smith et al., 2009, Young et al., 2016). Although recent studies reported GMVs alterations in multiple subcortical structures of MDD patients (Koolschijn et al., 2009, Lu et al., 2016, Nugent et al., 2013, Schmaal et al., 2016) and no significant GMVs alterations in subcortical regions of RD patients (Arnold et al., 2012), yet most studies did not include patients with RD, making it difficult to explore the potential subcortical MDD trait markers. The inclusion of RD patients is a key part of exploring the existence of potential trait markers of MDD in brain structure. For instance, Lu et al., (2016) found that, relative to healthy control (HC), first-episode drug-naïve MDD patients had significant GMVs reductions in the bilateral putamen and left thalamus, and speculated that the abnormalities of these two subcortical regions might be the potential trait markers of MDD. However, for the lack of RD group it was hard to speculate further whether these regions were the potential trait markers of MDD. Another important factor is that current MDD patients with a history of prior depressive episode and use of medication [e.g., either potential neurotoxic effects of prolonged disease or neuroprotective effects of ongoing medication (van Eijndhoven et al., 2009)] may obscure the identification of subcortical MDD trait markers. Previous researches have shown that the hippocampal GMV of MDD patients was significantly reduced compared to HC (Schmaal et al., 2016), especially if the individual has experienced persistent MDD for>2 years (McKinnon et al., 2009). Some studies in first-episode MDD patients found no significant GMVs alterations in hippocampus (Schmaal et al., 2016, van Eijndhoven et al., 2009). Regards to amygdala, the GMV studies have also shown inconsistency (Pagliaccio and Barch, 2016). A study showed that MDD patients (including first-episode/drug-naïve MDD patients) for the amygdalar GMV was significantly reduced (Bora et al., 2012, Sacher et al., 2012). However, it was also found that the GMV of the amygdala was significantly increased in the first-episode drug-naïve MDD patients (van Eijndhoven et al., 2009), or there was no significant GMV alteration in the amygdalar in MDD patients (Koolschijn et al., 2009, Schmaal et al., 2016). Additionally, some studies examining the thalamus GMVs in the MDD patients have also yielded conflicting results (Choi et al., 2020, Lu et al., 2016, Nugent et al., 2013, Sacchet et al., 2015, Schmaal et al., 2016, Wu et al., 2020). A study reported that (Schmaal et al., 2016) compared with HC, there was no significant alteration in the thalamic GMV of MDD patients. While the opposite result appeared in studies of first-episode (Lu et al., 2016) and drug-naïve MDD patients (Choi et al., 2020). In short, the inclusion of RD patients could provide a framework for exploring the potential subcortical MDD trait markers, studying the first-episode drug-naïve current MDD patients could provide an anchor point for identifying potential trait markers. Considering that the amygdala, hippocampus and thalamus play a very essential role in understanding the neural mechanism of depression (Disner et al., 2011, Mayberg, 1997, Phillips et al., 2003). Previous studies have only explored the whole GMV alterations of these three subcortical regions but not explored its subregion by themselves like exploring frontal subregions (e.g., DLPFC, VLPFC), which showed that the analysis of these three subcortical regions was not comprehensive. With the development of imaging analysis technology (Iglesias et al., 2018, Iglesias et al., 2015, Saygin et al., 2017), morphological studies based on the amygdalar, hippocampal and thalamic subregions have been applied to explore the potential neural mechanisms of disorders, such as Alzheimer's disease (Pardilla-Delgado et al., 2021), Parkinson's disease (Li et al., 2020), obsessive–compulsive disorder (Jurng et al., 2021, Weeland et al., 2021) and MDD (Brown et al., 2019, Kim et al., 2021, Yao et al., 2020). Exploration of the subregions in the thalamus, amygdala and hippocampus may provide more information to identify the potential subcortical MDD trait markers.

Additionally, negative stimulus processing requires participation of multiple subcortical regions then transmitting to upper cortical regions through the amygdala (Disner et al., 2011). Hyperactivity of bottom-up pathway may be experiencing altered network connections among subcortical structures that potentially influenced by regional subcortical GMV alterations. Whereas subcortical structure belongs to a highly interconnected network that supports integration of functionally diverse neural signal (Bell and Shine, 2016). Inter covariance of subcortical regions may provide an angle of view to understand the mechanism of potential trait and state marker. Covariance of brain areas reflects synchronized developmental changes in distributed brain regions (Alexander-Bloch et al., 2013, Mechelli et al., 2005), and thus represents co-operation among structures with coordinated maturation overlaid with plastic changes that develop in response to experience or degeneration (Palaniyappan et al., 2019). The application of graph theory to neuroimaging studies could yield useful quantitative metrics of global and regional topological properties, including structural covariance network (SCN) alterations involving subcortical structures. Structural covariance analysis has been used in studies examining potential brain mechanisms of MDD with a focus on exploring a potential SCN between frontal (extending to the cingulate) and amygdalar regions (Scheinost et al., 2018, Wang et al., 2016, Wu et al., 2017, Zuo et al., 2018). The SCN results obtained revealed similar regional differences of morphometric indices in corticolimbic structures. Subcortical-based SCN studies have been reported in Stroke (Wang et al., 2019) and autism spectrum disorder (Duan et al., 2020). Exploration of MDD-associated topological changes in networks involving highly interconnected subcortical regions may be useful for revealing mechanisms of MDD.

### 1.1 Aims of the study

The primary aim of the present study was to analyze structural MRI data from cMDD patients, RD patients, and age- and gender-matched HCs to detect trait (i.e. similar structural alterations observed in both cMDD and RD) markers of MDD in the subcortical structures. Given that related studies did not involve the subregions of the subcortical structures, the potential subcortical state-dependent (i.e. either structural alterations observed in cMDD or RD) markers would also be considered. Secondly, we would examine whether there may be MDD-associated alterations in subcortical networks. We hypothesized that trait and state-dependent markers of MDD may be observed within the caudate, putamen, NAc, pallidum, amygdala, hippocampus, and thalamic nuclei, and further hypothesized that subcortical SCN connectivity may be altered in both cMDD and RD patients.

## 2 Materials and methods

### 2.1 Participants

MDD patients were recruited from the outpatient population of the Second Xiangya Hospital, affiliated with Central South University in Changsha, China. HCs were recruited from two local colleges and from the Changsha community. All recruitment was from April 20, 2014 to Sep 16, 2018.

MDD was diagnosed independently by two well-trained psychiatrists using the Structured Clinical Interview for DSM-IV-TR Axis I Disorders-Patient Edition (First et al., 2002). For the cMDD group, the inclusion criteria were: (1) meeting the DSM-IV-TR criteria for a first MDD episode; (2) no prior episodes of depression and no signs of potential comorbidities; and (3) a score ≥ 18 on the 17-item Hamilton Depression Rating Scale (HAMD-17) (Hamilton, 1960, Zimmerman et al., 2013). For the RD group, the inclusion criteria were: (1) not meeting the DSM-IV-TR criteria for active MDD within the 30 days preceding MRI; (2) at least one episode of MDD within the past 10 years; and (3) a HAMD-17 score < 7. For the HC group, the inclusion criteria were: (1) no DSM-IV axis I disorder; and (2) a HAMD-17 score < 7 (Hamilton, 1960, Zimmerman et al., 2013). The exclusion criteria for all participants were: (1) any history of a DSM-IV-TR Axis I disorder (e.g., manic episodes with irritable mood, adjustment disorder with depressed mood, etc.), with the exception of MDD in the cMDD and RD groups; (2) a history of taking antidepressants or undergoing psychotherapy; (3) a history of alcohol or substance abuse; (4) any MRI contraindication; and (5) being pregnant or lactating, experiencing postpartum depression, or being in menopause.

This study protocol was approved by the Second Xiangya Hospital of Central South University’s Ethics Committee. All participants confirmed that they understood the purpose of our study and signed an informed consent form. A total of 434 participants (cMDD, N = 132; RD, N = 67; HC, N = 235, see details in supplementary Fig. S1) were enrolled in our study.

### 2.2 Assessments

In addition to HAMD-17 assessment, all participants completed a self-reported Rumination Response Scale (RRS) (Treynor et al., 2003) to quantitate ruminative level.

### 2.3 Image acquisition and processing

High-resolution images were obtained with a 3.0-T Magnetom Skyra scanner (Siemens Healthineers, Erlangen, Germany) equipped with a standard head coil and programmed with a three-dimensional T1-weighted magnetization-prepared rapid acquisition gradient echo sequence. In the MRI scanner, participants wore ear plugs to abate noise and fitted foam pads to prevent head motion. The image acquisition parameters were: 176 contiguous sagittal slices, 1900-ms repetition time, 2.01-ms echo time, 900-ms inversion time, 256 × 256 slice matrix, 1.0-mm slice thickness, 256 × 256-mm^2^ field of view, image voxel size of 1.0 × 1.0 × 1.0 mm^3^, 9° flip angle, and no gap.

Quality assurance (QA, see supplementary) framework of Computational Anatomy Toolbox (CAT) were used to check quality of original T1 image data (http://www.neuro.uni-jena.de/cat/index.html). FreeSurfer 7.1.1 software tools (http://surfer.nmr.mgh.harvard.edu/) were used to analyze T1 image data with in-program reconstruction procedures (Dale et al., 1999, Fischl, 2004, Fischl et al., 2002). Among them, MRI volumes were preprocessed (including segmentation) with volume-based streaming and subcortical tissue classes were labelled based on the basic algorithm used for cortical labeling (Fischl, 2004, Fischl et al., 2002).

FreeSurfer 7.1.1 software tools employed a probabilistic atlas built with ultra-high-resolution ex vivo MRI data to produce automated segmentation of amygdaloid (Saygin et al., 2017) and hippocampal (Iglesias et al., 2015) subfields; the approach was combined with histological data to produce thalamic (Iglesias et al., 2018) subfields. Manual method was used to correct inaccurate segmentation through visual inspection. Then reconstruction procedure was repeated until accurate segmentations were obtained. A command line with “asegstats2table” (https://surfer.nmr.mgh.harvard.edu/fswiki/asegstats2table) was used to extract GMVs of basal ganglia structures (i.e., the caudate, putamen, NAc, and pallidum), amygdaloid, hippocampal, thalamic subfields, and intracranial volumes were estimated based on the Fischl template (Fischl et al., 2002). Amygdaloid, hippocampal, and thalamic subfields were named in accordance with the aforementioned templates (regions and abbreviations were listed in Appendix A).

### 2.4 Structural covariance network construction and properties

**Fig. 1:**
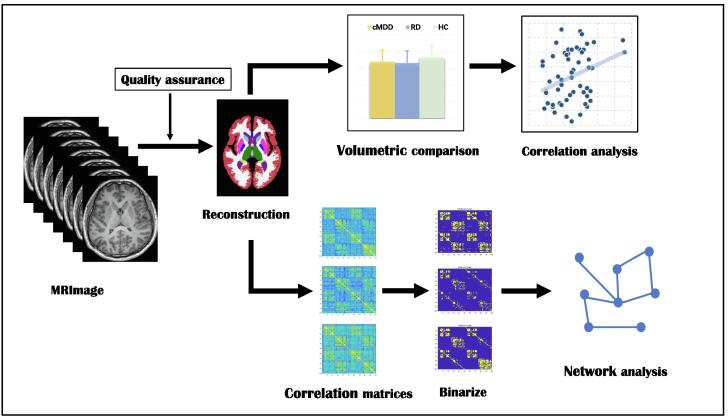
Summary of data preprocessing and analysis steps.

SCN construction and topological properties were computed with the use of the Graph Analysis Toolbox (Hosseini et al., 2012) based on the Brain Connectivity Toolbox (https://sites.google.com/site/bctnet/). We defined 100 subcortical regions of interest (ROIs), including the bilateral caudate, putamen, NAc, pallidum, 9 amygdaloid ROIs, 12 hippocampal ROIs, and 25 thalamic substructure ROIs (Table S1). ROIs were defined as SCN nodes, while linear correlations between ROI pairs were defined as edges, such that the correlation value *r_ij_* represented the correlation between structures *i* and *j*. We set age and intra-cranial volume as covariates and regressed them out (Walhovd et al., 2011), and then extracted regional GMV residuals. Because partial correlations could reduce SCN correlation transitivity but could not be computed when the number of ROIs exceeds the sample size [e.g. RD = 67, ROIs = 100; (Zalesky et al., 2012)], we used a 100 × 100 Pearson’s correlation matrix (*M*) of residuals of GMVs of the 100 subcortical ROIs to generate a binary adjacency matrix (if *r_ij_* exceeded a preset threshold, then *M_ij_* = 1; otherwise *M_ij_* = 0) for each group (whole brain-based measure). A minimum network density (*D_min_*) threshold was applied such that all nodes should be fully connected and not fragmented in the SCN (threshold range, 0.17–0.45, with increments of 0.02 and a fully connected graph at 0.16). Data preprocessing and analysis steps are summarized in Fig. 1.

ROI-based SCNs could be described and quantified based on the topological properties of characteristic path length (*L*), clustering coefficient (*C*), global efficiency (*E_global_*). Small-world network (*S*) architecture represented an optimal balance between local and global information processing. If *S* > 1, then the network of groups (cMDD, RD, and HC) was considered “small-world-ness” (Humphries and Gurney, 2008). Degree distribution represented specific characteristics of a network and its resilience to random failure attack. Random failure analysis was used to assess SCN resilience (Bullmore and Sporns, 2009). An optimization algorithm (Blondel et al., 2008, Newman, 2006) was used to find optimal modular structures (1000 iterations, Modularity) within each group’s network (Rubinov and Sporns, 2010). (For more details, see supplementary). Furthermore, we measured the centrality of each node (regional) between groups: degree (number of edges/node), clustering, and betweenness (fraction of all shortest paths in the network that pass-through a given node.) (Rubinov and Sporns, 2010).

### 2.5 Statistical analysis

#### 2.5.1 Clinical measurement

Inter-group differences in demographic and clinical characteristics were analyzed with Chi-square tests (gender), analyses of variance (ANOVAs; age, education and RRS score), and Kruskal-Wallis tests (HAMD-17 score). Duration of illness was compared between the cMDD and RD groups with a Mann-Whitney *U* test. The analyses were carried out in SPSS, version 26.0, with a significance criterion of *p* < 0.05 (2-tailed).

#### 2.5.2 Region of interest based between groups analyses and comparisons

For each subcortical ROI, multivariate analyses of covariance (MANCOVAs) were used to analyze inter-group volumetric differences, with each ROI volume treated as a dependent variable, participant group treated as a categorical predictor, and both age and intra-cranial volume treated as covariates. After determining whether there were main effects of group on ROI volumes, post-hoc *t*-tests were performed to compare the three groups pairwise. False discovery rate (FDR) for multiple comparisons was applied with2-tailed threshold *p* < 0.05.

#### 2.5.3 ROI-based network group comparisons

A non-parametric permutation test was used to assess inter-group differences (cMDD vs. HC; RD vs. HC; cMDD vs. RD) in topological properties (Bassett et al., 2008, Bernhardt et al., 2011). For each of a total of 1000 repetitions (Hosseini et al., 2013, 2012), the corrected GMV for each participant was reassigned randomly to one of two randomized groups with the same sample size as the original group; an association matrix was generated for each randomized group (*D_min_* = 0.17–0.45, 0.02 increments). Measurements of topological properties were calculated for all networks across all densities and then differences between the random groups were calculated (at each network density), resulting in a permutation distribution of difference under the null hypothesis. The real between-group difference in these measurements was placed in the corresponding permutation distribution and *p* values were calculated based on its percentile position (Bernhardt et al., 2011). We employed function data analysis (FDA), a non-parametric permutation test that compares curves across thresholds between groups (at threshold *p* < 0.05, 2-tailed). For results of regional topological property analyses, FDR correction for multiple comparisons was an applied 2-tailed threshold *p* < 0.05.

#### 2.5.4 ROI-psychometric correlation analyses

To explore whether potential trait or state markers correlated with psychometric traits (depressive symptom severity and ruminative level), Pearson (Illness remission and RRS scores) and Spearman (HAMD-17 scores and illness duration) correlational analyses were performed in SPSS 26.0 software with a significance threshold of *p* < 0.05 (2-tailed).

## 3 Results

### 3.1 Participant characteristics

Finally, a total of 433 participants (one removed from cMDD group, see supplementary) were enrolled in our study (Table 1). The cMDD, RD, and HC groups were confirmed to be similar with respect to gender, age, and years of education (Table 1). The cMDD group had higher HAMD-17, and RRS scores than the RD and HC groups (all *p* < 0.0001; Table 1).

**Table 1:** Demographic and clinical characteristics of participants by group.

Characteristic	cMDD(N = 131)	RD(N = 67)	HC(N = 235)	Statistic	
Gender, males/females	59/72	29/38	113/122	χ^2^ = 0.63	*p* = 0.73
Age, years	21.77 ± 4.01	21.70 ± 3.77	21.35 ± 2.91	F = 0.74	*p* = 0.48
Age range, years	18–35	18–35	18–44		
Education, years	14.00 ± 2.22	14.52 ± 2.22	14.25 ± 0.82	F = 1.83	*p* = 0.16
Education range, years	10–23	10–23	12–19		
Illness duration, months	9.46 ± 12.13	13.77 ± 8.98	–	U = 687.00	*p* < 0.0001
Illness remission, months	–	5.56 ± 3.63	–	–	–
HAMD-17 score	22.45 ± 4.88	3.72 ± 2.93	2.12 ± 2.31	H = 264.16	*p* < 0.0001
RRS score	58.29 ± 11.14	44.84 ± 10.19	43.95 ± 8.76	F = 90.87	*p* < 0.0001

Abbreviations: cMDD, first-episode drug-naïve currently depressed patients; RD, remitted MDD patients; HC, healthy control; HAMD-17, 17-item Hamilton Depression Rating Scale; RRS, Rumination Response Scale; χ^2^ , chi-square; F, analyses of variance; U, Mann-Whitney *U* test; H, Kruskal-Wallis test.

### 3.2 Volumetric differences

**Fig. 2:**
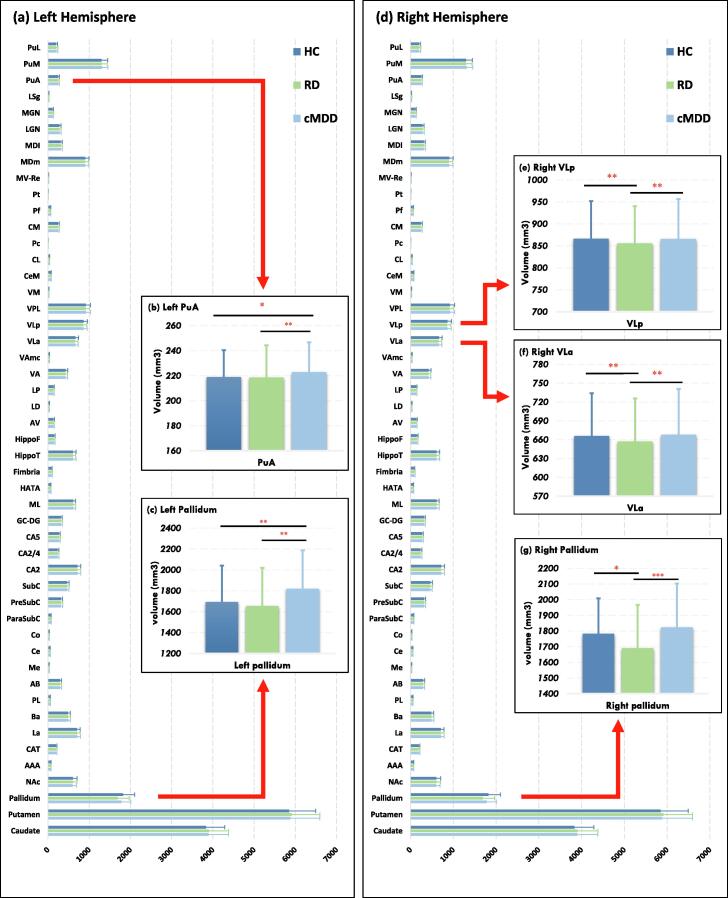
Volumetric differences among cMDD, RD, and HC groups (* *p* < 0.05; ** *p* < 0.01; *** *p* < 0.001).

Result showed a significant main effect of group on subcortical region GMVs (F = 2.27, *p* = 0.001; Wilk's λ = 0.88; partial η^2^ = 0.062). As shown in Table 2 and Fig. 2, the GMVs of the bilateral pallidum and thalamic subfields (left PuA, right VLa and right VLp) differed significantly among the three groups (*p* = 0.029 ∼ 0.041, FDR corrected; for more details, see supplementary Table S1). Post-hoc analysis revealed that, compared with HCs, both cMDD and RD patients had significantly decreased GMVs of the left pallidum (trait marker, Fig. 2c) and the left PuA (trait marker, Fig. 2b). Meanwhile, compared with HCs, RD patients had significantly decreased GMVs of the right pallidum (state-dependent marker, Fig. 2g). GMVs of the right VLa and the right VLp were significantly reduced (state-dependent marker, Fig. 2e and f) in RD patients, relative to HCs. Additionally, RD patients had significantly decreased GMVs of the right pallidum (Fig. 2g), the right VLa and the right VLp compared with cMDD patients (Fig. 2e and f).

**Table 2:** Significant Difference Subcortical GMVs among cMDD, RD, and HC groups, controlling for age and intra-cranial volume.

GMV region (dependent variable)	Hemi	cMDD		RD		HC		Statistic		
		(N = 131)		(N = 67)		(N = 235)				
		Mean	SD	Mean	SD	Mean	SD	F	FDR *p*	Partial η^2^
Pallidum	L	1693.50	348.33	1654.82	364.72	1819.17	368.50	8.06	0.029	0.024
	R	1783.30	225.63	1690.99	276.06	1823.81	278.50	7.44	0.029	0.034
Thalamus										
PuA	L	219.01	21.37	218.65	25.64	222.92	23.77	6.81	0.031	0.032
VLa	R	666.09	68.01	657.21	68.48	668.04	72.93	7.17	0.029	0.033
VLp	R	866.36	85.70	855.82	84.21	866.04	90.60	6.26	0.041	0.029

cMDD, first-episode drug-naïve currently depressed patients; RD, remitted MDD patients; HC, healthy control; Hemi, hemisphere; L, left hemi; R, right hemi; GMV, gray matter volume; SD, standard deviation; MANCOVA, multiple analyses of covariance; F, analyses of variance; η^2^, eta-square.

### 3.3 ROI-based structural covariance network group comparisons

With respect to global properties (Table 3 and supplementary Fig. S2-S6), the three groups showed small-world-ness (mean *S* across densities: cMDD = 1.16–1.18; RD = 1.33–1.36, and HC = 1.47). Both depressed patient groups had significantly lower *S* than HCs (both *p* = 0.001, FDA corrected). The cMDD group had lower *S* (*p* = 0.001, FDA corrected) and lower global efficiency (*p* = 0.04, FDA corrected) than the RD group. Conversely, both MDD patient groups had highly characteristic path length relative to HCs (*p* = 0.001, FDA corrected). Additionally, relative to HCs, the cMDD group had a significantly lower normalized path length γ (*p* = 0.012, FDA corrected) and modularity (*p* = 0.03, FDA corrected). The HC group had seven modules, as did the cMDD group, whereas the RD group had 14 modules. Between-group comparison between cMDD and RD patients demonstrated 7 and 10 modules, respectively (see supplementary Tables S2-S5). The networks of three groups followed an exponentially truncated power-law distribution of degree (Table 3 and supplementary Fig. S3).

**Table 3:** Comparisons of gray matter-based structural covariance network (SCN) topological properties between groups.

Global metrics	Group comparisons										
	cMDD vs. HC		*p*		RD vs. HC		*p*		cMDD vs. RD		*p*
Sigma (σ)	1.16	1.47	0.001		1.37	1.47	0.001		1.18	1.33	0.001
Gamma (γ)	1.37	1.79	0.012		1.48	1.80	0.46		1.39	1.49	0.17
Global efficiency	0.61	0.60	0.72		0.63	0.60	0.11		0.61	0.63	0.04
Characteristic path length	1.78	1.67	0.001		1.80	1.67	0.001		1.82	1.73	0.001
Lambda (λ)	1.06	1.02	0.001		1.05	1.02	0.001		1.07	1.03	0.001
modularity	0.30	0.40	0.03		0.30	0.40	0.87		0.30	0.30	0.12
Degree distribution											
Estimated exponent (*a*)	1.48	1.77			1.79	1.78			1.48	1.80	
Cut-off degree (*b*)	4.78	3.58			3.44	3.54			4.76	3.43	
R^2^	0.94	0.90			0.97	0.90			0.94	0.97	

Sigma, small-world-ness index; Gamma, normalized clustering coefficient; Lambda, normalized path length.

The R^2^ values obtained for distribution fit were 0.94 for the cMDD group, 0.97 for the RD group, and 0.90 for the HC, affirming good fitness. FDA-based results of our random failure analysis showed no significant differences between groups (cMDD vs. HCs, *p* = 0.19; RD vs. HCs, *p* = 0.09; cMDD vs. RD, *p* = 0.69; FDA corrected; see supplementary Fig. S5).

For regional properties (see supplementary Tables S6-S8), FDA-based centralities of clustering, degree, and betweenness comparisons were found to differ significantly across the right pallidum and subfields of the amygdala, hippocampus, and thalamus between the three groups. However, none of these differences survived correction for multiple comparisons (*p* > 0.05).

### 3.4 ROI-psychometric correlation analyses

The results of our correlation analysis of subcortical ROI GMVs with psychometric data are reported by group in Supplementary Table S9. Notably, for the RD group, RRS scores correlated directly with left pallidum GMVs (*r* = 0.29, *p* = 0.03) and there was a trend toward an inverse correlation between illness remission and left pallidum GMVs (*r* = -0.39, *p* = 0.07). Additionally, HAMD-17 scores of RD patients correlated inversely with right VLa, right VLp GMVs (*r* = -0.33 ∼ -0.30, *p* = 0.01 – 0.02).

## 4 Discussion

In the present study, we found altered left pallidum and left PuA GMVs in both cMDD and RD patients, relative to HCs, providing evidence of an MDD trait marker. Additionally, we found that patients in the RD group (but not in the cMDD group) had reduced GMVs of right pallidum, right VLa and right VLp, compared with HCs, providing evidence of a state-dependent marker. Structural covariance analysis showed that, compared to HCs, patients diagnosed with MDD had significant changes in small-world-ness index and path length values that were consistent with topological property trait-like markers of MDD that may be influenced by GMV alterations. We observed a correlation of structural alterations in the left pallidum of RD patients with RRS scores that may indicate an interaction between a presently presented potential trait marker and cognitive response style. Furthermore, the inverse correlation between GMVs of right thalamic subfields and HAMD-17 scores in RD patients may reflect relationships among residual symptoms, scar effects, and the presently presented potential state-dependent marker in the remitted depressive stage. These results may provide new insights relevant to all depressive stages with respect to the relationship between psychological behavior and structural alterations of brain regions.

### 4.1 Volumetric differences

One of key finding of this study was an apparent MDD trait marker of the left pallidum. Although, the pallidum seems not to be often considered in discussions of the neurobiological underpinnings of depression (Disner et al., 2011), it plays important roles in reward and motivation (Berridge and Kringelbach, 2015, Smith et al., 2009), and has been reported to be involved in emotional behavior (Drevets et al., 2008).

Structurally, the pallidum is divided into dorsal and ventral parts. The dorsal pallidum, consisting of the globus pallidus internus (GPi) and globus pallidus externus (GPe), is a key component of the cortico-basal ganglia-thalamocortical (CBGTC) circuit, which is important for motor system functioning (Silkis, 2001). The CBGTC circuit encompasses both the direct and indirect striatal output pathways. In the direct pathway, the striatum exerts an inhibitory action upon the GPi, thereby preventing it from inhibiting the thalamus, resulting in a more excitable thalamus that allows movements to be more easily initiated. In the indirect pathway, the striatum instead exerts an inhibitory action upon the GPe, preventing it from suppressing subthalamic nucleus-mediated excitation of the GPi. As a result, the indirect pathways acts to augment the subthalamic nucleus’ inhibitory influence on the thalamus, resulting in suppression of movement initiation (Silkis, 2001). The ventral pallidum is a core component of the limbic-cortical-striatal-pallidal-thalamic (LCSPT) circuit (Drevets et al., 2008), which is associated with the reward system (Berridge and Kringelbach, 2015). This impaired circuit appears to contribute to anhedonia (Berridge and Kringelbach, 2015, Russo and Nestler, 2013). Although NAc-prefrontal cortex circuitry (including the orbitofrontal cortex and medial prefrontal cortex) is a well characterized reward circuit (Russo and Nestler, 2013), Smith et al. (2009) have argued that the ventral pallidum is a major convergence point for reward signals, acting as an intermediary for cognitive, mood, and motor processing.

Furthermore, taken as a whole, the basal ganglia has a critical importance in the learning process. Previous studies showed that the basal ganglia were essential to “shifting to” a new set of rules and strengthen those new rules, while shifting from a new set of rules and learning new behavior-guiding rules were responsible for the frontal cortex (Ji et al., 2021, Owen et al., 1993, Wise and Rompre, 1989, Wise et al., 1996). Studies of cortical structure have reported decreased cortical thickness (or gyrification) of frontal cortex in depressed patients, representing trait markers (Xiong et al., 2019). Our results provided evidence that decreased volume of pallidum may contribute to break this switching process. Depressed patients would be hard to disengage from depressive state. Therefore, some degree of withdrawal behavior like (e.g. anhedonia) may be maintained in the remitted stage of MDD as a long term conservation of energy strategy (Beck and Bredemeier, 2016), even if RD patients may have fully remitted from a depressive mood.

Another evidence for this speculation was that rumination scores correlated directly with left pallidum GMV reductions in RD patients. Rumination level has been shown to be a positive prognostic factor for MDD recurrence risk (Buckman et al., 2018, Figueroa et al., 2019). The link between left pallidum GMV and rumination intensity may be an interaction point between a structural alteration of the brain and the neuropsychiatric processes underlying depressive episode recurrence risk. Therefore, reduced GMVs of the bilateral pallidum in RD patients may serve a protective function, promoting remission from residual symptoms. Furthermore, trait and state-dependent markers of MDD in subcortical structures may provide supporting evidence for the unified model of depression proposed by Beck and Bredemeir (2016), wherein a depressive episode was triggered when an individual who has lost a vital investment. Subsequently developed symptoms may serve as protective mechanisms for individual but maladaptive in contemporary times.

The presently observed thalamic subfield alteration (Iglesias et al., 2018), is another key finding. Notably, two studies found (Choi et al., 2020, Lu et al., 2016) decreased volume (and shape contraction) of the bilateral thalamus (especially in the dorsal and ventral parts) in first episode drug-naïve MDD patients, supporting our result. Within them, regional shape deformation of the left dorsal thalamus negatively correlated with HAMD score was also found in the study of Lu et al., (2016). The correlations that we observed for GMVs of ventral thalamic subfields with HAMD-17 scores (negative correlation) in RD patients. It suggested that the thalamus with different subregions could present current depressive severity in the diverse depressive stage. The thalamus, which is often described as a relay station that transmitted information between cerebral cortices and subcortical structures, has been implicated in biased processing of negative stimuli and reinforcement of self-referential schemas of depression (Disner et al., 2011). Especially, the pulvinar is responsible for selective visual attention. This region lesion would affect superior functions involving visual and language (Herrero et al., 2002). Trait marker of the PuA may be one of reason for depressed patients keeping biased cognitive pattern to process negative stimulus. Since the thalamus and the basal ganglia belong to an highly interconnected structure (Bell and Shine, 2016). Two trait markers (alterations in GMV of the pallidum and PuA) may represent the core issue of depressed patients is that negatively cognitive pattern which in turn strengthen the possibility of recurrence (Beck and Bredemeier, 2016, Disner et al., 2011).

Notably, Schmaal and van Velzen (2019) have suggested that MDD trait markers may be representative of a preexisting vulnerability factor, whereas state-dependent markers in the remitted state may be a scar effect that worsens with each new episode. Trait markers may be reflecting a persistent depressive vulnerability (or predisposition), whereas state-dependent markers of the remitted stage may constitute a new marker concept in MDD that is independent of the traditional conception of trait and state markers. However, this concept of scar effect still needs to be confirmed by more empirical studies, especially the influence of time course for the different depressive stage to brain structure.

It was also noteworthy that no significant GMVs differences were found in the hippocampus. Previous studies have shown that, relative to HC, the hippocampal GMVs of first-episode (Schmaal et al., 2016) and drug-naïve MDD patients (van Eijndhoven et al., 2009) did not changes significantly which supported the current results. Whereas, the significant reduction in the hippocampal GMV has been found in studies of patients with chronic or recurrent MDD (Frodl et al., 2008, Treadway et al., 2015). The *meta*-analysis of Kempton et al., (2011) found that the hippocampal GMV of RD patients was significantly larger than that of MDD patients, but there was no significant difference from HC. This indicated that the hippocampal volume may alter with changes in the depressive state. Recent studies have found that alterations of the dentate gyrus were related to the number of prior depressive episodes and stress level in MDD patients (Treadway et al., 2015), and increased GMV of the hippocampal tail was related to the remission of depressive symptoms (Maller et al., 2017). Therefore, the hippocampal volume alteration may be a biomarker of recurrent MDD rather than a trait (or state) marker of MDD (Belleau et al., 2019).

### 4.2 Subcortical covariance network differences

Aforementioned results provided evidence of subcortical trait and state-dependent markers of MDD. Although previous studies have shown alteration in structural covariance (Han et al., 2020, Wu et al., 2017, Zuo et al., 2018) and in cortical networks (Wang et al., 2016) in MDD, our study was the first, to our knowledge, to investigate the sophisticated relationship of subcortical trait and state-dependent markers with SCN alterations over different stages of depression. The SCN represents structural inter-relationships among different neuronal substrates, including both morphometric correlations and anatomical connectivities (Lim et al., 2013), that could be used to elucidate eloquent neurobiological mechanisms underlying structural neural networks (Bassett et al., 2008, Bernhardt et al., 2011). From this perspective, alterations of global properties may provide important information regarding the clinical significant of trait and state-dependent markers in MDD.

Our finding of small-world-ness in all three groups of this study indicated that we analyzed tightly linked subcortical structures, even across MDD stages. Our finding that degree distributions in the three groups all followed an exponentially truncated power law distribution indicated that there were a small number of SCN connections in many subcortical structures with a few structures having a large number of connections. Our findings of decreased GMVs of subcortical regions in both cMDD and RD groups, in the context of similar degree distributions of all three groups, indicating that significant deficits in topological properties may persist across MDD stages.

Compared with HCs, both of our MDD groups had lower small-world-ness and longer characteristic path lengths (and normalized path lengths). Generally, small-world networks were more clustered than random networks, but have approximately the same path length as random networks (Watts and Strogatz, 1998). Path lengths have been interpreted as estimates of the potential for functional integration between brain regions, such that a shorter path may imply a stronger potential for integration (Rubinov and Sporns, 2010). The presently observed alterations in small-world-ness and path lengths pointed to a reduction in subcortical structure inter-connectivity in MDD that may be influenced by trait and state-dependent markers of MDD. These alterations may contribute to the persistence of residual symptoms in the remitted state of depression.

Notably, compared with HCs, cMDD patients also had an altered modularity, a normalized clustering coefficient. There may be a specific set of alterations of multiple properties that were specific for active depression. For example, compared to our RD group, our cMDD group had significantly lower small-world-ness and global efficiency values together with a longer characteristic path length. These alterations may impact structural connectivity of depressed patients and, eventually, contribute to maladaptive hyperactivation of corresponding brain functions.

Energy conservation, manifested as anhedonia (and/or psychomotor symptoms), may be a core mechanism of depression (Beck and Bredemeier, 2016). Meanwhile, Altered SCN connectivity in the remitted stage of depression may also reflect part of scar effect mechanism (Schmaal and van Velzen, 2019), with RD patients maintaining a core cognitive style of energy conservation that is characteristic of depressiveness, and this maintained effect may reflect a recurrence risk factor (Monroe et al., 2019). Although altered topological SCN properties of cortical regions have been reported in a previous study of depression (Wang et al., 2016), the present study only focused on the subcortical structures. Trait marker, state-dependent marker, and SCN data for subcortical structures thus would provide new evidence to contribute to the elucidation of neurobiological mechanisms of depression (Beck and Bredemeier, 2016, Disner et al., 2011, Drevets et al., 2008, Young et al., 2016).

In general, there were volumetric differences between the groups for the pallidum and the thalamic subregions, but the differences in the FDA-based node centralities were mainly in the subfields of the amygdala, hippocampus, and thalamus (although none of the node centralities survived correction for multiple comparison). We speculated that the local morphological alterations of the potential subcortical MDD trait (or state-dependent) markers may induce changes in the node centralities of the corresponding regions, which in turn affect the changes in the node centralities of the adjacent subcortical structures, although the adjacent subcortical structures may not show significant morphological alterations. These alterations may eventually contribute to the subcortical SCN changes in different stages of depression. From our results, the exploration for brain structural-based the complex relationship between morphology and topological properties may provide information for understanding the complex relationship between structural and functional network (Honey et al., 2007, Rubinov and Sporns, 2010) in the neurobiological mechanisms of depression (Disner et al., 2011, Pagliaccio and Barch, 2016, Young et al., 2016). Although previous studies showed that structure and function were highly correlated, this correspondence was imperfect (Honey et al., 2009) because function reflects the complex multi-synaptic interactions in the structural network (Suárez et al., 2020). Thus, a large number of empirical studies were in need to clarify the transition between brain structural and functional network in MDD.

Regarding study limitations, the presently suggested subcortical structure trait marker should be investigated further to tease out the multiple distinct morphometric properties incorporated within the GMV parameter (Ho et al., 2020). Secondly, we did not include SCN analysis of cortical structures, although cortical trait markers of MDD have been presented in recent studies (van Eijndhoven et al., 2013, Xiong et al., 2019). Cortical trait markers may provide more evidence of SCN abnormalities related to different stages of depression and may provide insights into the sequence of symptom re-emergence in the onset of recurrence. Future studies should explore alterations of functional networks over different stages of MDD in relation to the presence of trait and state markers, including trait and state markers revealed in previous functional imaging studies (Cheng et al., 2019, Dong et al., 2019b, Dong et al., 2019a, Ming et al., 2017). Last but not least, regarding the purpose and key findings of trait markers, the number of prior episodes of RD group were not restricted; therefore, it remains unclear whether a history of prior depressive episodes could induce and manifest some brain structure alterations itself. Though previous studies have shown that some brain structures in depressed patients may not be affected by number of prior depressive episodes (Arnold et al., 2012, Treadway et al., 2015), the emerging view on theory of trait and state marker of MDD have introduced scar effects (Allott et al., 2016, Schmaal and van Velzen, 2019). This effect does not exist before the onset of the disease but appears during remission and gets worse with each new episode, like a scar. This possible effect is impossible to address with comparisons in the current dataset, which may still need more longitude empirical observations.

## 5 Conclusions

The present findings support the supposition that MDD ha characteristic underlying trait and state-dependent markers in the pallidum and thalamic subfields, and suggest that altered topological properties of subcortical SCNs show trait-like topological features. The presently reported direct correlation of rumination scores with decreased left pallidum GMVs in RD patients may reflect an interaction point between a brain structural alteration and one’s neuropsychiatric risk of depression recurrence. These results may be useful for therapy planning, targeting of interventions for MDD and the knowledge of core mechanism of depression. For example, Belge et al. (2020) found that patients with MDD had a significantly increased pallidum (holistic) volume after electroconvulsive therapy (ECT) and this effect correlated inversely with agitation improvement.

## Funding

This work was supported by the National Natural Science Foundation of China [Grant number 82071532].

### CRediT authorship contribution statement

**Ge Xiong:** Data curation, Formal analysis, Investigation, Writing – original draft, Writing – review & editing. **Daifeng Dong:** Data curation, Investigation, Writing – review & editing. **Chang Cheng:** Data curation, Investigation, Writing – review & editing. **Yali Jiang:** Data curation, Investigation, Writing – review & editing. **Xiaoqiang Sun:** Data curation, Investigation, Writing – review & editing. **Jiayue He:** Data curation, Investigation, Writing – review & editing. **Chuting Li:** Data curation, Investigation, Writing – review & editing. **Yidian Gao:** Data curation, Investigation, Writing – review & editing. **Xue Zhong:** Data curation, Investigation, Writing – review & editing. **Haofei Zhao:** Data curation, Investigation, Writing – review & editing. **Xiang Wang:** Writing – review & editing. **Shuqiao Yao:** Conceptualization, Investigation, Writing – review & editing.

## Declaration of Competing Interest

The authors declare that they have no known competing financial interests or personal relationships that could have appeared to influence the work reported in this paper.

## Figures and Tables

**Table:** 

Region of Interests	Abbreviation
Caudate	–
Putamen	–
Nucleus Accumbens	NAc
Pallidum	–
*Amygdaloid Subfields*	
Anterior Amygdaloid Area	AAA
Cortico amygdaloid Transition Area	CAT
Lateral Nucleus	La
Basal Nucleus	Ba
Paralaminar Nucleus	PL
Accessory Basal	AB
Medial	Me
Central	Ce
Cortical	Co
*Hippocampal Subfields*	
Parasubiculum	ParaSubC
Presubiculum	PreSubC
Subiculum	SubC
Cornu Ammonis 1	CA1
Cornu Ammonis 2/3	CA2/3
Cornu Ammonis 4	CA4
Granule Cell Layer of Dentate Gyrus	GC-DG
Molecular Layer	ML
Hippocampus Amygdala Transition Area	HATA
Fimbria	–
Hippocampal Tail	HippoT
Hippocampal Fissure	HippoF
*Thalamic Subfields*	
Anteroventral	AV
Laterodorsal	LD
Lateral Posterior	LP
Ventral Anterior	VA
Ventral Anterior Magnocellular	VAmc
Ventral Lateral Anterior	VLa
Ventral Lateral Posterior	VLp
Ventral Posterolateral	VPL
Ventromedial	VM
Central Medial	CeM
Central Lateral	CL
Paracentral	Pc
Centromedian	CM
Parafascicular	Pf
Paratenial	Pt
Reuniens (Medial Ventral)	MV-Re
Mediodorsal Lateral Magnocellular	MDm
Mediodorsal Lateral Parvocellular	MDl
Lateral Geniculate	LGN
Medial Geniculate	MGN
Limitans (Suprageniculate)	LSg
Pulvinar Anterior	PuA
Pulvinar Medial	PuM
Pulvinar Lateral	PuL
Pulvinar Inferior	PuI
