# Supplementary material for: Potential structural trait markers of depression in the form of alterations in the structures of subcortical nuclei and structural covariance network properties
Source: Neuroimage Clin. 2021 Nov 3;32:102871. doi: 10.1016/j.nicl.2021.102871 (PMC8578037; doi:10.1016/j.nicl.2021.102871)
Supplement: Supplementary data 1 [file mmc1.docx]

**Potential structural trait markers of depression in the form of alterations in the structures of subcortical nuclei and structural covariance network properties**

**Supplementary**

Ge Xiong ^a,b^, Daifeng Dong ^a,b^, Chang Cheng ^a,b^, Yali Jiang ^a,b,d^, Xiaoqiang Sun ^a,b^, Jiayue He ^a,b^, Chuting Li ^a,b,c^, Yidian Gao ^a,b^, Xue Zhong ^a,b^, Haofei Zhao ^a,b^, Xiang Wang ^a,b,c^, Shuqiao Yao ^a,b,c,⁎^

^a^ Medical Psychological Center, The Second Xiangya Hospital, Central South University, Changsha, Hunan 410011, China

^b^ Medical Psychological Institute of Central South University, Changsha, Hunan 410011, China

^c^ China National Clinical Research Center on Mental disorders (Xiangya), Changsha, Hunan 410011, China

^d^ School of Psychology, South China Normal University, Guangzhou 510631, China

^⁎^ Corresponding author at: Medical Psychological Center, The Second Xiangya Hospital, Central South University, Changsha, Hunan 410011, China.

E-mail address: [shuqiaoyao@csu.edu.cn](mailto:shuqiaoyao@csu.edu.cn)

**Content:**

**1.** Quality assurance

**2.** Topological properties of structural covariance network

**2.1** Small-world-ness

**2.2** Modularity

**2.3** Degree distribution

**2.4** Random failure analysis

**3.** Result of quality assurance

**Table S1** MANCOVA of parts of Subcortical Regions among cMDD, RD, and HC groups, controlling for age and intra-cranial volume.

**Table S2** Modularity in cMDD patients.

**Table S3** Modularity 1 in RD patients.

**Table S4** Modularity 2 in RD patients.

**Table S5** Modularity in Healthy controls.

**Table S6** Regional Topological Properties between cMDD patients and HC.

**Table S7** Regional Topological Properties between RD patients and HC.

**Table S8** Regional Topological Properties between cMDD and RD patients.

**Table S9** Pearson and Spearman Correlations of Two Depressed Groups.

**Fig. S1** Recruitment flow chart for all participants.

**Fig. S2** Differences of (a, b, c) Sigma (small-world-ness index), (d, e, f) Global efficiency, (g, h, i) Gamma (normalized clustering coefficient) and (j, k, l) Modularity among the three Groups.

**Fig. S3** Result of the degree distribution in (a, d, g) cMDD, (b, e, h) RD and (c, f, i) HC groups.

**Fig. S4** Differences of Characteristic Path Length and Lamba (normalized path length) among the three Groups.

**Fig. S5** Results of random attack among the three Groups.

**Fig. S6** Changes in Global Network Measures as a Function of Network Density (Sigma: small-world-ness; Gamma: normalized clustering coefficient; Lambda: normalized path length).

**1. Quality assurance**

Preprocessing of MRI strongly depends on the quality of the original input data. Especially multi-center studies and data-sharing projects need to consider varying image properties due to different scanners, sequences and protocols. Therefore, CAT produces this retrospective quality assurance (QA) framework for empirical quantification of quality differences in different scans or studies. This QA framework allows the evaluation of essential image parameters such as noise, inhomogeneities and image resolution. All quality measures would be scaled to a rating scale which easily allows to compare measures across different scanners and sequences (Fig. S-QA1).


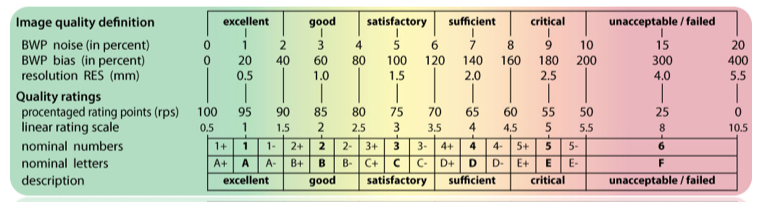


**Fig. S-QA1** The Rating Scale of Image Quality Measurements (<http://www.neuro.uni-jena.de/cat/index.html#QA>).

Based on the rating scale of image quality measurements, image parameters (resolution, noise and bias, see Fig. S-QA2) should be at least ‘B-’ in each image data that we included.


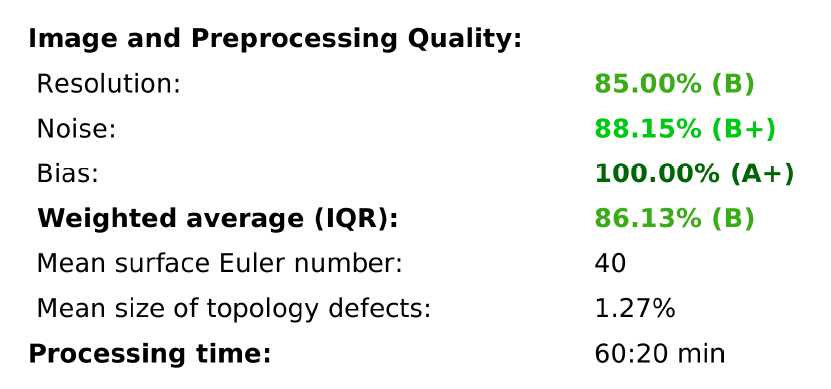


**Fig. S-QA2** Example of Result of Image Quality.

**2. Topological properties of structural covariance network**

ROI-based SCNs can be described and quantified based on the topological properties of integration (characteristic path length, *L*) and segregation (clustering coefficient, *C*). Integration reflects a network’s ability for global information processing. Segregation represents the ability of a network to process information locally. Therefore, *L* represents the shortest path length between all node pairs. The average *L* between all pairs of nodes in the network produces matrix of *L* (*ML*). The inverse of *ML*, global efficiency (*E_global_*) represents efficiency of information transfer. The value of *C* represents the degree of covariance (number of edges) among nodes. The average C of nodes produces a C matrix (*MC*). We measured the centrality of each node between groups: degree (number of edges/node), clustering, and betweenness (fraction of all shortest paths in the network that pass-through a given node.) (Rubinov and Sporns, 2010).

**2.1 Small-world-ness**

Small-world network architecture represents an optimal balance between local and global information processing. As described previously (Hosseini et al., 2013; Palaniyappan et al., 2019; Rubinov and Sporns, 2010), *ML* and *MC* were compared to corresponding mean values of a benchmark random graph (*ML_random_* and *MC_random_*) to evaluate inter-group small-world index values, that is, *S* values (cMDD vs. HC; RD vs. HC; or cMDD vs. RD) (Maslov and Sneppen, 2002; Milo et al., 2002), where *ML_random_* and *MC_random_* were generated based on the mean *C* and *L* values of *m* random networks and *m* is the number of null networks generated for normalization of clustering and path length (*m* = 20 in our study). Thus, *S* = [(*MC*/*MC_random_*)/(*ML*/*ML_random_*)]. If *S* > 1, then the network of groups (cMDD, RD, and HC) was considered “small-world-ness” (Humphries and Gurney, 2008).

**2.2 Modularity**

An optimization algorithm (Blondel et al., 2008; Newman, 2006) was used to find optimal modular structures (1000 iterations) within each group’s network. Modularity, a sophisticated measure of network segregation (Rubinov and Sporns, 2010), was quantified by subdividing the network into groups of regions (nodes) with maximal within-group links and minimal between-group links (Girvan and Newman, 2002).

**2.3 Degree distribution**

We measured degree distribution between groups. Degree distribution represents specific characteristics of a network and its resilience to random failure attack. Degree distributions of small-world SCNs follow the following exponentially truncated power-law distribution:

*P(d) ~* [*d ^(a-1)^ * exp ^(-d/b)^*]

where *P(d)* is the probability of the network regional degree *d*, *a* is the scaling regimen exponent, and *b* is the cut-off degree above which there is an exponential decay in hub probability. R^2^ value was used as a fitting index of how well the cumulative degree distribution of the constructed network followed an exponentially truncated power-law distribution; an R^2^ value of 1 would indicate a perfect fit.

**2.4 Random failure analysis**

Random failure analysis was used to assess SCN resilience (Bullmore and Sporns, 2009). The random failure of networks was simulated by randomly removing one node from the network and measuring changes in global network metrics of the remaining largest component. Each step of the random node removal procedure was repeated 100 times, and the procedure was repeated until the size of the largest components was 1 (Achard et al., 2006).

**3. Result of quality assurance**

Result showed all image data had a quality at least ‘good’ except for one from cMDD group (Table S-QA1).

**Table S-QA1** Result of Image Quality for all Participants

| **Image parameter** | **Resolution** | **Noise** | **Bias** |
| --- | --- | --- | --- |
| Inclusion (N=433) |  |  |  |
| Quality definition (%) | 85 | 81.43 ~ 90.24 | 100 |
| Quality rating | B | B- ~ A- | A+ |
| Exclusion (N=1) |  |  |  |
| Quality definition (%) | 85 | 74.35 | 100 |
| Quality rating | B | C | A+ |

**References**

Achard, S., Salvador, R., Whitcher, B., Suckling, J., Bullmore, E., 2006. A Resilient, Low-Frequency, Small-World Human Brain Functional Network with Highly Connected Association Cortical Hubs. J. Neurosci. 26, 63. https://doi.org/10.1523/JNEUROSCI.3874-05.2006

Blondel, V.D., Guillaume, J.-L., Lambiotte, R., Lefebvre, E., 2008. Fast unfolding of communities in large networks. Journal of Statistical Mechanics: Theory and Experiment 2008, P10008. https://doi.org/10.1088/1742-5468/2008/10/p10008

Bullmore, E., Sporns, O., 2009. Complex brain networks: graph theoretical analysis of structural and functional systems. Nature Reviews Neuroscience 10, 186–198. https://doi.org/10.1038/nrn2575

Girvan, M., Newman, M.E.J., 2002. Community structure in social and biological networks. Proc Natl Acad Sci USA 99, 7821. https://doi.org/10.1073/pnas.122653799

Hosseini, S.M.H., Black, J.M., Soriano, T., Bugescu, N., Martinez, R., Raman, M.M., Kesler, S.R., Hoeft, F., 2013. Topological properties of large-scale structural brain networks in children with familial risk for reading difficulties. NeuroImage 71, 260–274. https://doi.org/10.1016/j.neuroimage.2013.01.013

Humphries, M.D., Gurney, K., 2008. Network ‘Small-World-Ness’: A Quantitative Method for Determining Canonical Network Equivalence. PLOS ONE 3, e0002051. https://doi.org/10.1371/journal.pone.0002051

Maslov, S., Sneppen, K., 2002. Specificity and Stability in Topology of Protein Networks. Science 296, 910. https://doi.org/10.1126/science.1065103

Milo, R., Shen-Orr, S., Itzkovitz, S., Kashtan, N., Chklovskii, D., Alon, U., 2002. Network Motifs: Simple Building Blocks of Complex Networks. Science 298, 824. https://doi.org/10.1126/science.298.5594.824

Newman, M.E.J., 2006. Modularity and community structure in networks. Proc Natl Acad Sci USA 103, 8577. https://doi.org/10.1073/pnas.0601602103

Palaniyappan, L., Hodgson, O., Balain, V., Iwabuchi, S., Gowland, P., Liddle, P., 2019. Structural covariance and cortical reorganisation in schizophrenia: a MRI-based morphometric study. Psychol. Med. 49, 412–420. https://doi.org/10.1017/S0033291718001010

Rubinov, M., Sporns, O., 2010. Complex network measures of brain connectivity: Uses and interpretations. NeuroImage 52, 1059–1069. https://doi.org/10.1016/j.neuroimage.2009.10.003

Table S1 MANCOVA of parts of Subcortical Regions among cMDD, RD, and HC groups, controlling for age and intra-cranial volume.

| **GMV region (dependent variable)** | **Hemi** | **cMDD** | |  | **RD** | |  | **HC** | |  | **Statistic** | | |
| --- | --- | --- | --- | --- | --- | --- | --- | --- | --- | --- | --- | --- | --- |
|  |  | **(N = 131)** | |  | **(N = 67)** | |  | **(N = 235)** | |  |  |  |  |
|  |  | **Mean** | **SD** |  | **Mean** | **SD** |  | **Mean** | **SD** |  | **F** | **FDR *p*** | **Partial** **η^2^** |
| Caudate | L | 3811.83 | 491.50 |  | 3766.11 | 433.66 |  | 3761.70 | 455.80 |  | 1.52 | 0.51 | 0.007 |
|  | R | 3897.11 | 482.08 |  | 3904.45 | 478.33 |  | 3837.62 | 449.50 |  | 0.67 | 0.67 | 0.003 |
| Putamen | L | 6040.11 | 833.12 |  | 6018.80 | 872.48 |  | 5882.44 | 763.25 |  | 1.58 | 0.51 | 0.008 |
|  | R | 5882.83 | 710.45 |  | 5916.76 | 678.08 |  | 5850.79 | 643.77 |  | 0.55 | 0.69 | 0.003 |
| NAc | L | 590.50 | 110.15 |  | 593.33 | 115.83 |  | 590.38 | 106.82 |  | 0.48 | 0.70 | 0.002 |
|  | R | 598.72 | 90.62 |  | 608.88 | 96.10 |  | 605.97 | 99.86 |  | 0.75 | 0.67 | 0.004 |
| *Amygdaloid subfields* |  |  |  |  |  |  |  |  |  |  |  |  |  |
| AAA | L | 61.28 | 7.67 |  | 59.91 | 7.77 |  | 60.86 | 8.11 |  | 2.62 | 0.35 | 0.012 |
|  | R | 66.91 | 8.37 |  | 66.51 | 7.99 |  | 65.77 | 8.17 |  | 0.91 | 0.65 | 0.004 |
| CAT | L | 199.22 | 26.98 |  | 202.78 | 27.05 |  | 197.87 | 26.00 |  | 0.03 | 0.97 | <0.0001 |
|  | R | 195.92 | 22.57 |  | 196.67 | 23.51 |  | 196.40 | 22.11 |  | 0.78 | 0.67 | 0.004 |
| La | L | 696.78 | 74.96 |  | 686.75 | 67.36 |  | 693.81 | 78.90 |  | 3.06 | 0.29 | 0.014 |
|  | R | 715.74 | 72.12 |  | 708.20 | 75.52 |  | 706.93 | 74.83 |  | 2.05 | 0.44 | 0.010 |
| Ba | L | 479.55 | 55.20 |  | 475.94 | 53.63 |  | 476.42 | 52.50 |  | 2.16 | 0.44 | 0.010 |
|  | R | 494.29 | 51.68 |  | 491.81 | 56.01 |  | 488.51 | 53.28 |  | 1.11 | 0.59 | 0.005 |
| PL | L | 55.14 | 6.53 |  | 54.66 | 6.37 |  | 54.59 | 6.28 |  | 2.08 | 0.44 | 0.010 |
|  | R | 54.39 | 5.56 |  | 54.20 | 6.49 |  | 53.80 | 6.02 |  | 0.87 | 0.65 | 0.004 |
| AB | L | 286.17 | 36.13 |  | 288.49 | 36.01 |  | 285.33 | 34.13 |  | 0.76 | 0.67 | 0.004 |
|  | R | 294.95 | 34.78 |  | 293.75 | 36.25 |  | 294.33 | 33.67 |  | 1.57 | 0.51 | 0.007 |
| Me | L | 22.53 | 5.41 |  | 22.46 | 5.38 |  | 22.70 | 5.09 |  | 0.91 | 0.65 | 0.004 |
|  | R | 24.33 | 6.21 |  | 24.08 | 5.70 |  | 24.41 | 5.36 |  | 1.07 | 0.63 | 0.005 |
| Ce | L | 44.16 | 7.51 |  | 44.91 | 7.90 |  | 45.36 | 8.25 |  | 2.14 | 0.44 | 0.010 |
|  | R | 49.45 | 7.91 |  | 48.66 | 8.46 |  | 48.50 | 8.50 |  | 1.26 | 0.55 | 0.006 |
| Co | L | 28.79 | 4.68 |  | 29.14 | 4.79 |  | 28.50 | 4.42 |  | 0.22 | 0.83 | 0.001 |
|  | R | 29.74 | 4.55 |  | 29.84 | 5.30 |  | 29.79 | 4.03 |  | 0.97 | 0.64 | 0.005 |
| *Hippocampal subfields* |  |  |  |  |  |  |  |  |  |  |  |  |  |
| ParaSubC | L | 72.33 | 11.62 |  | 72.43 | 10.89 |  | 73.03 | 12.01 |  | 0.57 | 0.69 | 0.003 |
|  | R | 69.05 | 11.47 |  | 68.83 | 10.38 |  | 67.88 | 10.64 |  | 0.56 | 0.69 | 0.003 |
| PreSubC | L | 332.01 | 42.07 |  | 337.44 | 43.60 |  | 334.47 | 41.13 |  | 0.44 | 0.74 | 0.002 |
|  | R | 317.71 | 36.36 |  | 318.69 | 38.06 |  | 317.71 | 35.30 |  | 0.37 | 0.76 | 0.002 |
| SubC | L | 470.29 | 48.25 |  | 475.79 | 56.69 |  | 469.13 | 50.99 |  | 0.11 | 0.92 | 0.001 |
|  | R | 464.41 | 43.20 |  | 467.96 | 49.53 |  | 465.15 | 47.61 |  | 0.52 | 0.70 | 0.002 |
| CA1 | L | 683.81 | 72.24 |  | 684.43 | 74.89 |  | 681.69 | 74.53 |  | 0.82 | 0.67 | 0.004 |
|  | R | 720.54 | 75.81 |  | 714.35 | 76.88 |  | 717.57 | 76.83 |  | 1.93 | 0.44 | 0.009 |
| CA2/3 | L | 222.61 | 27.44 |  | 221.62 | 27.79 |  | 219.54 | 27.52 |  | 0.71 | 0.67 | 0.003 |
|  | R | 239.33 | 29.32 |  | 236.92 | 31.33 |  | 235.14 | 29.59 |  | 1.30 | 0.55 | 0.006 |
| CA4 | L | 264.00 | 24.99 |  | 265.26 | 28.20 |  | 262.33 | 24.96 |  | 0.32 | 0.77 | 0.002 |
|  | R | 273.03 | 25.03 |  | 273.16 | 28.73 |  | 270.90 | 25.55 |  | 0.67 | 0.67 | 0.003 |
| GC-DG | L | 309.42 | 28.98 |  | 311.41 | 32.85 |  | 308.27 | 29.90 |  | 0.38 | 0.74 | 0.002 |
|  | R | 317.66 | 28.64 |  | 318.34 | 32.99 |  | 315.65 | 29.25 |  | 0.66 | 0.67 | 0.003 |
| ML | L | 597.77 | 54.93 |  | 599.08 | 58.68 |  | 594.89 | 55.17 |  | 0.74 | 0.67 | 0.004 |
|  | R | 617.55 | 54.29 |  | 615.13 | 56.33 |  | 613.03 | 56.54 |  | 1.35 | 0.54 | 0.006 |
| HATA | L | 64.44 | 9.74 |  | 65.42 | 8.99 |  | 64.65 | 11.07 |  | 0.18 | 0.85 | 0.001 |
|  | R | 64.19 | 10.43 |  | 62.99 | 9.84 |  | 63.15 | 9.15 |  | 1.20 | 0.56 | 0.006 |
| Fimbria | L | 95.59 | 20.25 |  | 96.41 | 25.12 |  | 91.96 | 19.79 |  | 0.99 | 0.64 | 0.005 |
|  | R | 86.44 | 17.51 |  | 88.21 | 20.52 |  | 83.37 | 15.88 |  | 1.26 | 0.55 | 0.006 |
| HippoT | L | 582.74 | 66.82 |  | 586.57 | 56.69 |  | 580.14 | 63.56 |  | 0.06 | 0.95 | <0.0001 |
|  | R | 610.20 | 72.04 |  | 609.36 | 61.71 |  | 609.23 | 73.57 |  | 0.58 | 0.69 | 0.003 |
| HippoF | L | 149.65 | 23.70 |  | 151.02 | 25.95 |  | 151.55 | 24.91 |  | 0.60 | 0.69 | 0.003 |
|  | R | 150.18 | 22.76 |  | 149.97 | 24.36 |  | 150.25 | 24.95 |  | 0.27 | 0.80 | 0.001 |
| *Thalamic subfields* |  |  |  |  |  |  |  |  |  |  |  |  |  |
| AV | L | 125.89 | 20.92 |  | 127.12 | 17.45 |  | 128.69 | 21.11 |  | 1.30 | 0.55 | 0.006 |
|  | R | 135.12 | 18.66 |  | 131.67 | 17.12 |  | 135.85 | 21.70 |  | 2.89 | 0.31 | 0.014 |
| LD | L | 26.02 | 9.22 |  | 24.97 | 7.28 |  | 26.94 | 8.97 |  | 1.84 | 0.44 | 0.009 |
|  | R | 27.13 | 9.40 |  | 25.66 | 7.39 |  | 28.80 | 9.67 |  | 3.96 | 0.19 | 0.019 |
| LP | L | 138.03 | 21.80 |  | 136.00 | 21.56 |  | 137.93 | 20.19 |  | 1.46 | 0.52 | 0.007 |
|  | R | 131.36 | 20.54 |  | 129.23 | 19.29 |  | 132.00 | 20.71 |  | 1.77 | 0.44 | 0.008 |
| VA | L | 436.62 | 51.23 |  | 440.12 | 51.72 |  | 442.21 | 55.28 |  | 2.37 | 0.39 | 0.011 |
|  | R | 428.27 | 47.69 |  | 425.92 | 47.03 |  | 429.93 | 49.94 |  | 3.77 | 0.19 | 0.018 |
| VAmc | L | 33.36 | 4.50 |  | 33.47 | 4.10 |  | 33.69 | 4.55 |  | 1.87 | 0.44 | 0.009 |
|  | R | 34.87 | 3.83 |  | 34.53 | 3.68 |  | 35.21 | 4.28 |  | 4.68 | 0.14 | 0.022 |
| VLa | L | 646.77 | 67.91 |  | 647.71 | 72.67 |  | 650.00 | 72.28 |  | 2.71 | 0.34 | 0.013 |
|  | R | (see Table 2) | |  |  |  |  |  |  |  |  |  |  |
| VLp | L | 837.84 | 80.75 |  | 837.78 | 88.89 |  | 838.99 | 85.63 |  | 2.66 | 0.34 | 0.013 |
|  | R | (see Table 2) | |  |  |  |  |  |  |  |  |  |  |
| VPL | L | 841.59 | 90.02 |  | 845.74 | 97.68 |  | 847.19 | 100.12 |  | 1.79 | 0.44 | 0.008 |
|  | R | 927.29 | 106.19 |  | 914.08 | 93.95 |  | 921.99 | 109.31 |  | 4.44 | 0.14 | 0.021 |
| VM | L | 19.55 | 2.90 |  | 19.70 | 2.94 |  | 19.86 | 3.12 |  | 2.06 | 0.44 | 0.010 |
|  | R | 23.16 | 3.37 |  | 22.37 | 2.61 |  | 22.77 | 3.69 |  | 4.73 | 0.14 | 0.022 |
| CeM | L | 66.91 | 9.41 |  | 67.05 | 9.30 |  | 67.01 | 8.55 |  | 0.65 | 0.67 | 0.003 |
|  | R | 70.64 | 8.55 |  | 69.27 | 8.27 |  | 70.42 | 8.94 |  | 2.69 | 0.34 | 0.013 |
| CL | L | 33.70 | 6.86 |  | 33.43 | 5.74 |  | 32.83 | 7.00 |  | 1.78 | 0.44 | 0.008 |
|  | R | 34.54 | 6.10 |  | 33.67 | 5.93 |  | 34.90 | 6.65 |  | 2.32 | 0.40 | 0.011 |
| Pc | L | 4.09 | 0.55 |  | 4.03 | 0.54 |  | 4.01 | 0.54 |  | 1.90 | 0.44 | 0.009 |
|  | R | 4.68 | 0.64 |  | 4.66 | 0.63 |  | 4.69 | 0.60 |  | 1.51 | 0.51 | 0.007 |
| CM | L | 238.45 | 24.70 |  | 240.05 | 27.53 |  | 236.40 | 27.11 |  | 0.50 | 0.70 | 0.002 |
|  | R | 248.28 | 26.54 |  | 246.42 | 27.38 |  | 245.51 | 29.64 |  | 2.39 | 0.39 | 0.011 |
| Pf | L | 54.62 | 8.56 |  | 54.80 | 7.59 |  | 53.47 | 10.55 |  | 0.37 | 0.75 | 0.002 |
|  | R | 58.66 | 9.60 |  | 58.21 | 9.14 |  | 57.30 | 11.35 |  | 0.72 | 0.70 | 0.003 |
| Pt | L | 6.45 | 0.77 |  | 6.53 | 0.84 |  | 6.45 | 0.80 |  | 0.48 | 0.70 | 0.002 |
|  | R | 7.40 | 0.84 |  | 7.38 | 0.86 |  | 7.36 | 0.89 |  | 1.77 | 0.44 | 0.008 |
| MV-Re | L | 12.69 | 2.34 |  | 12.79 | 2.54 |  | 12.94 | 2.05 |  | 1.26 | 0.55 | 0.006 |
|  | R | 13.88 | 2.25 |  | 13.83 | 2.64 |  | 13.95 | 2.38 |  | 0.71 | 0.70 | 0.003 |
| MDm | L | 866.99 | 95.34 |  | 862.97 | 113.11 |  | 879.86 | 92.07 |  | 4.39 | 0.14 | 0.021 |
|  | R | 902.30 | 91.78 |  | 898.50 | 87.32 |  | 908.66 | 88.47 |  | 3.40 | 0.22 | 0.016 |
| MDI | L | 296.76 | 35.17 |  | 298.65 | 43.44 |  | 302.66 | 32.89 |  | 3.81 | 0.19 | 0.018 |
|  | R | 313.06 | 37.17 |  | 313.08 | 33.49 |  | 312.27 | 33.56 |  | 1.15 | 0.58 | 0.005 |
| LGN | L | 257.01 | 34.10 |  | 255.22 | 35.20 |  | 261.79 | 34.81 |  | 3.41 | 0.22 | 0.016 |
|  | R | 286.40 | 34.32 |  | 285.07 | 35.85 |  | 282.43 | 34.20 |  | 0.97 | 0.64 | 0.005 |
| MGN | L | 109.76 | 17.47 |  | 112.22 | 19.37 |  | 105.87 | 21.00 |  | 1.55 | 0.51 | 0.007 |
|  | R | 113.86 | 18.47 |  | 117.90 | 22.26 |  | 112.30 | 20.82 |  | 0.44 | 0.73 | 0.002 |
| LSg | L | 26.23 | 8.18 |  | 28.52 | 8.45 |  | 26.30 | 7.83 |  | 0.77 | 0.67 | 0.004 |
|  | R | 20.28 | 6.49 |  | 22.19 | 6.67 |  | 19.93 | 5.72 |  | 1.40 | 0.54 | 0.007 |
| PuA | L | (see Table 2) | |  |  |  |  |  |  |  |  |  |  |
|  | R | 251.47 | 25.65 |  | 249.66 | 25.12 |  | 251.30 | 27.71 |  | 3.54 | 0.21 | 0.017 |
| PuM | L | 1111.17 | 124.21 |  | 1096.52 | 141.14 |  | 1112.01 | 129.24 |  | 3.73 | 0.19 | 0.018 |
|  | R | 1313.34 | 140.94 |  | 1302.16 | 136.22 |  | 1299.94 | 147.92 |  | 1.99 | 0.44 | 0.009 |
| PuL | L | 163.87 | 28.57 |  | 169.73 | 30.72 |  | 166.27 | 26.89 |  | 0.67 | 0.67 | 0.003 |
|  | R | 203.29 | 32.91 |  | 208.77 | 36.01 |  | 198.46 | 33.96 |  | 0.89 | 0.65 | 0.004 |
| PuI | L | 236.98 | 31.15 |  | 237.48 | 32.55 |  | 237.82 | 33.54 |  | 0.56 | 0.69 | 0.003 |
|  | R | 294.65 | 39.84 |  | 296.74 | 42.35 |  | 286.84 | 43.10 |  | 1.36 | 0.54 | 0.006 |

cMDD, first-episode drug-naïve currently depressed patients; RD, remitted MDD patients; HC, healthy control; Hemi, hemisphere; L, left hemi; R, right hemi; GMV, gray matter volume; SD, standard deviation; MANCOVA, multivariate analyses of covariance; F, analyses of variance; η^2^, eta-square.

Table S2 Modularity in cMDD patients.

| Module 1 | Module 2 | Module 3 | Module 4 | Module 5 | Module 6 | Module 7 |
| --- | --- | --- | --- | --- | --- | --- |
| L_Pal | L_ AV | L_AAA | L_Put | L_Cau | L_HippoF | L_NAc |
| R_Pal | R_AV | R_AAA | R_Put | R_Cau | R_HippoF | R_NAc |
|  | L_LD | L_CAT |  |  |  |  |
|  | R_LD | R_CAT |  |  |  |  |
|  | L_ LP | L_La |  |  |  |  |
|  | R_LP | R_La |  |  |  |  |
|  | L_VA | L_Ba |  |  |  |  |
|  | R_VA | R_Ba |  |  |  |  |
|  | L_VAmc | L_PL |  |  |  |  |
|  | R_VAmc | R_PL |  |  |  |  |
|  | L_VLa | L_AB |  |  |  |  |
|  | R_VLa | R_AB |  |  |  |  |
|  | L_VLp | L_Me |  |  |  |  |
|  | R_VLp | R_Me |  |  |  |  |
|  | L_VPL | L_Ce |  |  |  |  |
|  | R_VPL | R_Ce |  |  |  |  |
|  | L_VM | L_Co |  |  |  |  |
|  | R_VM | R_Co |  |  |  |  |
|  | L_CeM | L_ParaSubC |  |  |  |  |
|  | R_CeM | R_ParaSubC |  |  |  |  |
|  | L_CL | L_PresSubC |  |  |  |  |
|  | R_CL | R_PresSubC |  |  |  |  |
|  | L_Pc | L_SubC |  |  |  |  |
|  | R_Pc | R_SubC |  |  |  |  |
|  | L_CM | L_CA1 |  |  |  |  |
|  | R_CM | R_CA2 |  |  |  |  |
|  | L_Pf | L_CA2/3 |  |  |  |  |
|  | R_Pf | R_CA2/4 |  |  |  |  |
|  | L_Pt | L_CA4 |  |  |  |  |
|  | R_Pt | R_CA5 |  |  |  |  |
|  | L_MV-Re | L_GC-DG |  |  |  |  |
|  | R_MV-Re | R_GC-DG |  |  |  |  |
|  | L_MDm | L_ML |  |  |  |  |
|  | R_MDm | R_ML |  |  |  |  |
|  | L_MDl | L_HATA |  |  |  |  |
|  | R_MDl | R_HATA |  |  |  |  |
|  | L_LGN | L_Fimbria |  |  |  |  |
|  | R_LGN | R_Fimbria |  |  |  |  |
|  | L_MGN | L_HippoT |  |  |  |  |
|  | R_MGN | R_HippoT |  |  |  |  |
|  | L_LSg |  |  |  |  |  |
|  | R_LSg |  |  |  |  |  |
|  | L_PuA |  |  |  |  |  |
|  | R_PuA |  |  |  |  |  |
|  | L_PuM |  |  |  |  |  |
|  | R_PuM |  |  |  |  |  |
|  | L_PuL |  |  |  |  |  |
|  | R_PuL |  |  |  |  |  |
|  | L_PuI |  |  |  |  |  |
|  | R_PuI |  |  |  |  |  |

L, Left hemisphere; R, Right hemisphere; cMDD, first-episode drug-naïve currently depressed patients.

Table S3 Modularity 1 in RD patients ^a^.

| Module 1 | Module 2 | Module 3 | Module 4 | Module 5 | Module 6 | Module 7 | Module 8 | Module 9 | Module 10 | Module 11 | Module 12 | Module 13 | Module 14 |
| --- | --- | --- | --- | --- | --- | --- | --- | --- | --- | --- | --- | --- | --- |
| L_VPL | L_AAA | L_Cau | L_Ce | R_Me | L_Pal | L_NAc | L_HippoF | R_Fimbria | L_HippoT | L_Put | L_AV | L_LD | R_VPL |
| R_VPL | R_AAA | R_Cau | R_Ce | R_Co | R_Pal | R_NAc | R_HippoF |  | R_HippoT | R_Put | R_AV | R_LD | R_VM |
| L_VM | L_CAT |  |  |  |  |  |  |  |  |  | L_VA | L_LP | R_CM |
| L_CM | R_CAT |  |  |  |  |  |  |  |  |  | R_VA | R_LP | L_Pf |
| L_Pt | L_La |  |  |  |  |  |  |  |  |  | L_VAmc | L_CL | R_Pf |
| L_MDm | R_La |  |  |  |  |  |  |  |  |  | R_VAmc | R_CL | R_Pt |
| L_MDl | L_Ba |  |  |  |  |  |  |  |  |  | L_VLa |  | R_LGN |
| L_LGN | R_Ba |  |  |  |  |  |  |  |  |  | R_VLa |  | L_MGN |
| L_PuA | L_PL |  |  |  |  |  |  |  |  |  | L_VLp |  | R_MGN |
| L_PuM | R_PL |  |  |  |  |  |  |  |  |  | R_VLp |  | L_LSg |
| L_PuI | L_AB |  |  |  |  |  |  |  |  |  | L_CeM |  | R_LSg |
|  | R_AB |  |  |  |  |  |  |  |  |  | R_CeM |  | R_PuA |
|  | L_Me |  |  |  |  |  |  |  |  |  | L_Pc |  | R_PuM |
|  | L_Co |  |  |  |  |  |  |  |  |  | R_Pc |  | R_PuI |
|  | L_ParaSubC |  |  |  |  |  |  |  |  |  | L_MV-Re |  |  |
|  | R_ParaSubC |  |  |  |  |  |  |  |  |  | R_MV-Re |  |  |
|  | L_PresSubC |  |  |  |  |  |  |  |  |  | R_MDm |  |  |
|  | R_PresSubC |  |  |  |  |  |  |  |  |  |  |  |  |
|  | L_SubC |  |  |  |  |  |  |  |  |  |  |  |  |
|  | R_SubC |  |  |  |  |  |  |  |  |  |  |  |  |
|  | L_CA1 |  |  |  |  |  |  |  |  |  |  |  |  |
|  | R_CA1 |  |  |  |  |  |  |  |  |  |  |  |  |
|  | L_CA2/3 |  |  |  |  |  |  |  |  |  |  |  |  |
|  | R_CA2/3 |  |  |  |  |  |  |  |  |  |  |  |  |
|  | L_CA4 |  |  |  |  |  |  |  |  |  |  |  |  |
|  | R_CA4 |  |  |  |  |  |  |  |  |  |  |  |  |
|  | L_GC-DG |  |  |  |  |  |  |  |  |  |  |  |  |
|  | R_GC-DG |  |  |  |  |  |  |  |  |  |  |  |  |
|  | L_ML |  |  |  |  |  |  |  |  |  |  |  |  |
|  | R_ML |  |  |  |  |  |  |  |  |  |  |  |  |
|  | L_HATA |  |  |  |  |  |  |  |  |  |  |  |  |
|  | R_HATA |  |  |  |  |  |  |  |  |  |  |  |  |
|  | L_Fimbria |  |  |  |  |  |  |  |  |  |  |  |  |

^a^ for RD vs. HC patients; L, Left hemisphere; R, Right hemisphere; RD, remitted MDD patients; HC, healthy control.

Table S4 Modularity 2 in RD patients ^a^.

| Module 1 | Module 2 | Module 3 | Module 4 | Module 5 | Module 6 | Module 7 | Module 8 | Module 9 | Module 10 |
| --- | --- | --- | --- | --- | --- | --- | --- | --- | --- |
| L_AV | L_Cau | L_LD | R_VPL | L_VPL | L_NAc | L_Put | L_HippoT | L_Pal | L_HippoF |
| R_AV | R_Cau | R_LD | R_VM | L_VM | R_NAc | R_Put | R_HippoT | R_Pal | R_HippoF |
| L_VA | L_AAA | L_LP | L_CM | L_Pt |  |  |  |  |  |
| R_VA | R_AAA | R_LP | R_CM | L_MDm |  |  |  |  |  |
| L_VAmc | L_CAT | L_CL | L_Pf | R_MDm |  |  |  |  |  |
| R_VAmc | R_CAT | R_CL | R_Pf | L_MDl |  |  |  |  |  |
| L_VLa | L_La |  | R_Pt | R_MDl |  |  |  |  |  |
| R_VLa | R_La |  | R_LGN | L_LGN |  |  |  |  |  |
| L_VLp | L_Ba |  | L_MGN | L_PuA |  |  |  |  |  |
| R_VLp | R_Ba |  | R_MGN | L_PuM |  |  |  |  |  |
| L_CeM | L_PL |  | L_LSg | L_PuI |  |  |  |  |  |
| R_CeM | R_PL |  | R_LSg |  |  |  |  |  |  |
| L_Pc | L_AB |  | R_PuA |  |  |  |  |  |  |
| R_Pc | R_AB |  | R_PuM |  |  |  |  |  |  |
| L_MV-Re | L_Me |  | L_PuL |  |  |  |  |  |  |
| R_MV-Re | R_Me |  | R_PuL |  |  |  |  |  |  |
|  | L_Ce |  | R_PuI |  |  |  |  |  |  |
|  | R_Ce |  |  |  |  |  |  |  |  |
|  | L_Co |  |  |  |  |  |  |  |  |
|  | R_Co |  |  |  |  |  |  |  |  |
|  | L_ParaSubC |  |  |  |  |  |  |  |  |
|  | R_ParaSubC |  |  |  |  |  |  |  |  |
|  | L_PreSubC |  |  |  |  |  |  |  |  |
|  | R_PreSubC |  |  |  |  |  |  |  |  |
|  | L_SubC |  |  |  |  |  |  |  |  |
|  | R_SubC |  |  |  |  |  |  |  |  |
|  | L_CA1 |  |  |  |  |  |  |  |  |
|  | R_CA1 |  |  |  |  |  |  |  |  |
|  | L_CA2/3 |  |  |  |  |  |  |  |  |
|  | R_CA2/3 |  |  |  |  |  |  |  |  |
|  | L_CA4 |  |  |  |  |  |  |  |  |
|  | R_CA4 |  |  |  |  |  |  |  |  |
|  | L_GC-DG |  |  |  |  |  |  |  |  |
|  | R_GC-DG |  |  |  |  |  |  |  |  |
|  | L_ML |  |  |  |  |  |  |  |  |
|  | R_ML |  |  |  |  |  |  |  |  |
|  | L_HATA |  |  |  |  |  |  |  |  |
|  | R_HATA |  |  |  |  |  |  |  |  |
|  | L_Fimbria |  |  |  |  |  |  |  |  |
|  | R_Fimbria |  |  |  |  |  |  |  |  |

^a^ for cMDD vs. RD patients; L, Left hemisphere; R, Right hemisphere; cMDD, first-episode drug-naïve currently depressed patients; RD, remitted MDD patients.

Table S5 Modularity in Healthy controls.

| Module 1 | Module 2 | Module 3 | Module 4 | Module 5 | Module 6 | Module 7 |
| --- | --- | --- | --- | --- | --- | --- |
| L_Put | R_AV | L_AV | L_AAA | L_Pal | L_Cau | L_HippoF |
| R_Put | R_LP | L_LD | R_AAA | R_Pal | R_Cau | R_HippoF |
| L_NAc | R_VA | R_LD | L_CAT |  |  |  |
| R_NAc | R_VLa | L_LP | R_CAT |  |  |  |
|  | R_VLp | L_VA | L_La |  |  |  |
|  | R_VPL | L_VAmc | R_La |  |  |  |
|  | R_VM | L_VLa | L_Ba |  |  |  |
|  | R_CeM | L_VLp | R_Ba |  |  |  |
|  | R_CL | L_VPL | L_PL |  |  |  |
|  | R_Pc | L_VM | R_PL |  |  |  |
|  | R_CM | L_CeM | L_AB |  |  |  |
|  | L_Pf | L_CL | R_AB |  |  |  |
|  | R_Pf | L_Pc | L_Me |  |  |  |
|  | R_Pt | L_CM | R_Me |  |  |  |
|  | R_MV-Re | L_Pt | L_Ce |  |  |  |
|  | R_MDm | L_MV-Re | R_Ce |  |  |  |
|  | R_MDl | L_MDm | L_Co |  |  |  |
|  | R_LGN | L_MDl | R_Co |  |  |  |
|  | L_MGN | L_LGN | L_ParaSubC |  |  |  |
|  | R_MGN | L_PuA | R_ParaSubC |  |  |  |
|  | L_LSg | L_PuM | L_PresSubC |  |  |  |
|  | R_LSg | L_PuI | R_PresSubC |  |  |  |
|  | R_PuA |  | L_SubC |  |  |  |
|  | R_PuM |  | R_SubC |  |  |  |
|  | L_PuL |  | L_CA1 |  |  |  |
|  | R_PuL |  | R_CA2 |  |  |  |
|  | R_PuI |  | L_CA2/3 |  |  |  |
|  |  |  | R_CA2/4 |  |  |  |
|  |  |  | L_CA4 |  |  |  |
|  |  |  | R_CA5 |  |  |  |
|  |  |  | L_GC-DG |  |  |  |
|  |  |  | R_GC-DG |  |  |  |
|  |  |  | L_ML |  |  |  |
|  |  |  | R_ML |  |  |  |
|  |  |  | L_HATA |  |  |  |
|  |  |  | R_HATA |  |  |  |
|  |  |  | L_Fimbria |  |  |  |
|  |  |  | R_Fimbria |  |  |  |
|  |  |  | L_HippoT |  |  |  |
|  |  |  | R_HippoT |  |  |  |

L, Left hemisphere; R, Right hemisphere.

Table S6 Regional Topological Properties between cMDD patients and HC.

| **Regional metrics** | **Directions** | **FDA *p*** | **FDR *p*** |
| --- | --- | --- | --- |
| ***Clustering centrality*** |  |  |  |
| L_CAT | cMDD > HC | 0.005 | 0.200 |
| L_Ba | cMDD < HC | 0.019 | 0.270 |
| L_AB | cMDD < HC | 0.006 | 0.200 |
| L_ML | cMDD < HC | 0.035 | 0.438 |
| L_MGN | cMDD > HC | 0.042 | 0.467 |
| L_LSg | cMDD < HC | 0.013 | 0.217 |
| R_Pal | cMDD < HC | 0.047 | 0.470 |
| R_VM | cMDD > HC | 0.012 | 0.217 |
| R_Pf | cMDD < HC | 0.013 | 0.217 |
| R_LSg | cMDD < HC | 0.002 | 0.200 |
| ***Degree centrality*** |  |  |  |
| L_CAT | cMDD > HC | 0.018 | 0.475 |
| L_AB | cMDD > HC | 0.001 | 0.100 |
| L_CA1 | cMDD > HC | 0.049 | 0.475 |
| L_Pf | cMDD < HC | 0.047 | 0.475 |
| L_LSg | cMDD < HC | 0.034 | 0.475 |
| R_Pf | cMDD < HC | 0.034 | 0.475 |
| R_Pt | cMDD > HC | 0.047 | 0.475 |
| R_LSg | cMDD < HC | 0.045 | 0.475 |
| ***Betweenness centrality*** |  |  |  |
| L_CAT | cMDD > HC | 0.018 | 0.360 |
| L_Ba | cMDD < HC | 0.038 | 0.556 |
| L_AB | cMDD > HC | 0.004 | 0.200 |
| R_AAA | cMDD < HC | 0.002 | 0.200 |
| R_Co | cMDD < HC | 0.012 | 0.300 |
| R_VA | cMDD > HC | 0.007 | 0.233 |
| R_MGN | cMDD > HC | 0.046 | 0.556 |

L, Left hemisphere; R, Right hemisphere; cMDD, first-episode drug-naïve currently depressed patients; HC, healthy control.

Table S7 Regional Topological Properties between RD patients and HC.

| **Regional metrics** | **Directions** | **FDA *p*** | **FDR *p*** |
| --- | --- | --- | --- |
| ***Clustering centrality*** |  |  |  |
| L_VPL | RD > HC | 0.045 | 0.775 |
| L_CL | RD < HC | 0.016 | 0.775 |
| L_MDI | RD > HC | 0.046 | 0.775 |
| R_HippoT | RD < HC | 0.035 | 0.775 |
| ***Degree centrality*** |  |  |  |
| L_Fimbria | RD > HC | 0.005 | 0.350 |
| L_HippoT | RD < HC | 0.042 | 0.631 |
| L_VA | RD > HC | 0.035 | 0.631 |
| L_LSg | RD > HC | 0.007 | 0.350 |
| R_Co | RD < HC | 0.012 | 0.400 |
| ***Betweenness centrality*** |  |  |  |
| L_Ba | RD > HC | 0.039 | 0.557 |
| L_AB | RD > HC | 0.036 | 0.557 |
| L_VA | RD > HC | 0.036 | 0.557 |
| L_CM | RD < HC | 0.036 | 0.557 |
| R_AAA | RD < HC | 0.014 | 0.557 |
| R_Co | RD < HC | 0.008 | 0.557 |
| R_VLp | RD < HC | 0.031 | 0.557 |

L, Left hemisphere; R, Right hemisphere; RD, remitted MDD patients; HC, healthy control.

Table S8 Regional Topological Properties between cMDD and RD patients.

| **Regional metrics** | **Directions** | **FDA *p*** | **FDR *p*** |
| --- | --- | --- | --- |
| ***Clustering centrality*** |  |  |  |
| L_CL | cMDD > RD | 0.002 | 0.200 |
| L_PuA | cMDD < RD | 0.028 | 0.729 |
| R_Fimbria | cMDD > RD | 0.04 | 0.729 |
| R_HippoT | cMDD > RD | 0.046 | 0.729 |
| R_VLp | cMDD > RD | 0.025 | 0.729 |
| ***Degree centrality*** |  |  |  |
| L_Co | cMDD > RD | 0.004 | 0.167 |
| L_Fimbria | cMDD < RD | 0.044 | 0.489 |
| L_Pf | cMDD < RD | 0.036 | 0.488 |
| L_LSg | cMDD < RD | 0.001 | 0.100 |
| L_PuA | cMDD > RD | 0.039 | 0.488 |
| R_CAT | cMDD > RD | 0.031 | 0.489 |
| R_Fimbria | cMDD < RD | 0.019 | 0.475 |
| R_Pf | cMDD < RD | 0.035 | 0.489 |
| R_PuL | cMDD < RD | 0.005 | 0.167 |
| ***Betweenness centrality*** |  |  |  |
| R_VLp | cMDD < RD | 0.001 | 0.100 |
| R_VM | cMDD < RD | 0.018 | 0.762 |
| R_Pul | cMDD < RD | 0.023 | 0.762 |

L, Left hemisphere; R, Right hemisphere; cMDD, first-episode drug-naïve currently depressed patients; HC, healthy control.

Table S9 Pearson and Spearman Correlations of Two Depressed Groups.

| **ROIs** | **Correlation coefficients (*p*)** | | | |
| --- | --- | --- | --- | --- |
|  | Illness duration | Illness remission | HAMD-17 score | RRS score |
| *cMDD patients* |  |  |  |  |
| L_Pallidum | 0.06 (0.58) | - | -0.05 (0.57) | -0.01 (0.90) |
| R_Pallidum | 0.09 (0.37) | - | 0.01 (0.93) | -0.07 (0.42) |
| L_PuA | 0.08 (0.47) | - | -0.02 (0.81) | -0.003 (0.97) |
| R_VLa | 0.04 (0.72) | - | -0.09 (0.31) | 0.002 (0.99) |
| R_VLp | 0.07 (0.48) | - | -0.10 (0.25) | 0.01 (0.92) |
| *RD patients* |  |  |  |  |
| L_Pallidum | 0.13 (0.51) | *-0.39 (0.07)* | -0.01 (0.92) | **0.29 (0.03)** |
| R_Pallidum | -0.06 (0.77) | -0.26 (0.24) | -0.02 (0.86) | 0.12 (0.39) |
| L_PuA | 0.08 (0.72) | -0.15 (0.52) | -0.12 (0.37) | 0.19 (0.14) |
| R_VLa | -0.25 (0.21) | 0.07 (0.75) | **-0.33 (0.01)** | 0.09 (0.52) |
| R_VLp | -0.28 (0.17) | 0.02 (0.94) | **-0.30 (0.02)** | 0.09 (0.53) |

cMDD, first-episode drug-naïve currently depressed patients; HC, healthy control; HAMD-17, the 17-item Hamilton Depression Rating Scale; RRS, Rumination Response Scale.


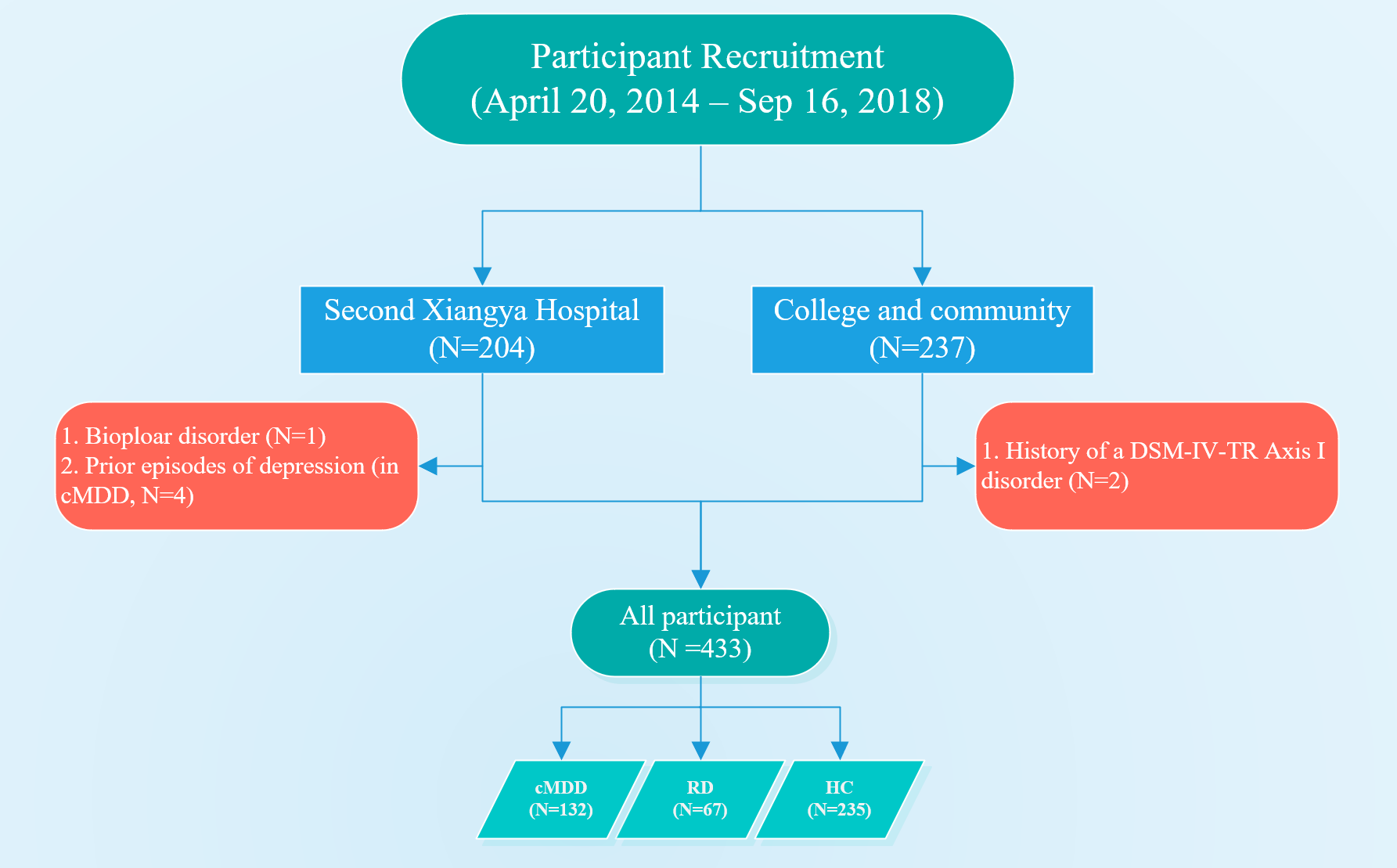


Fig. S1 Recruitment flow chart for all participants.


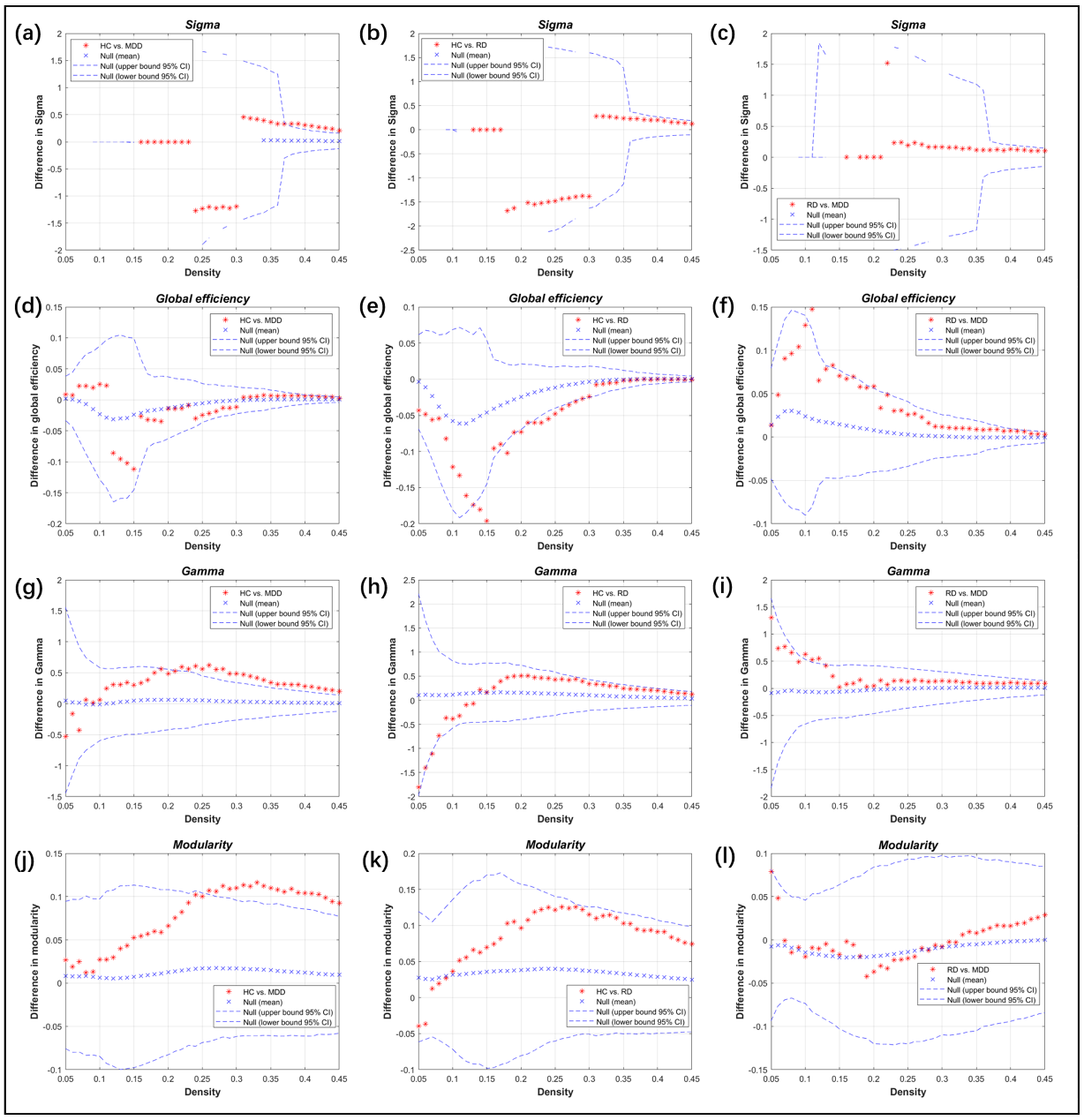


Fig. S2 Differences of (a, b, c) Sigma (small-world-ness index), (d, e, f) Global efficiency, (g, h, i) Gamma (normalized clustering coefficient) and (j, k, l) Modularity among the three Groups.


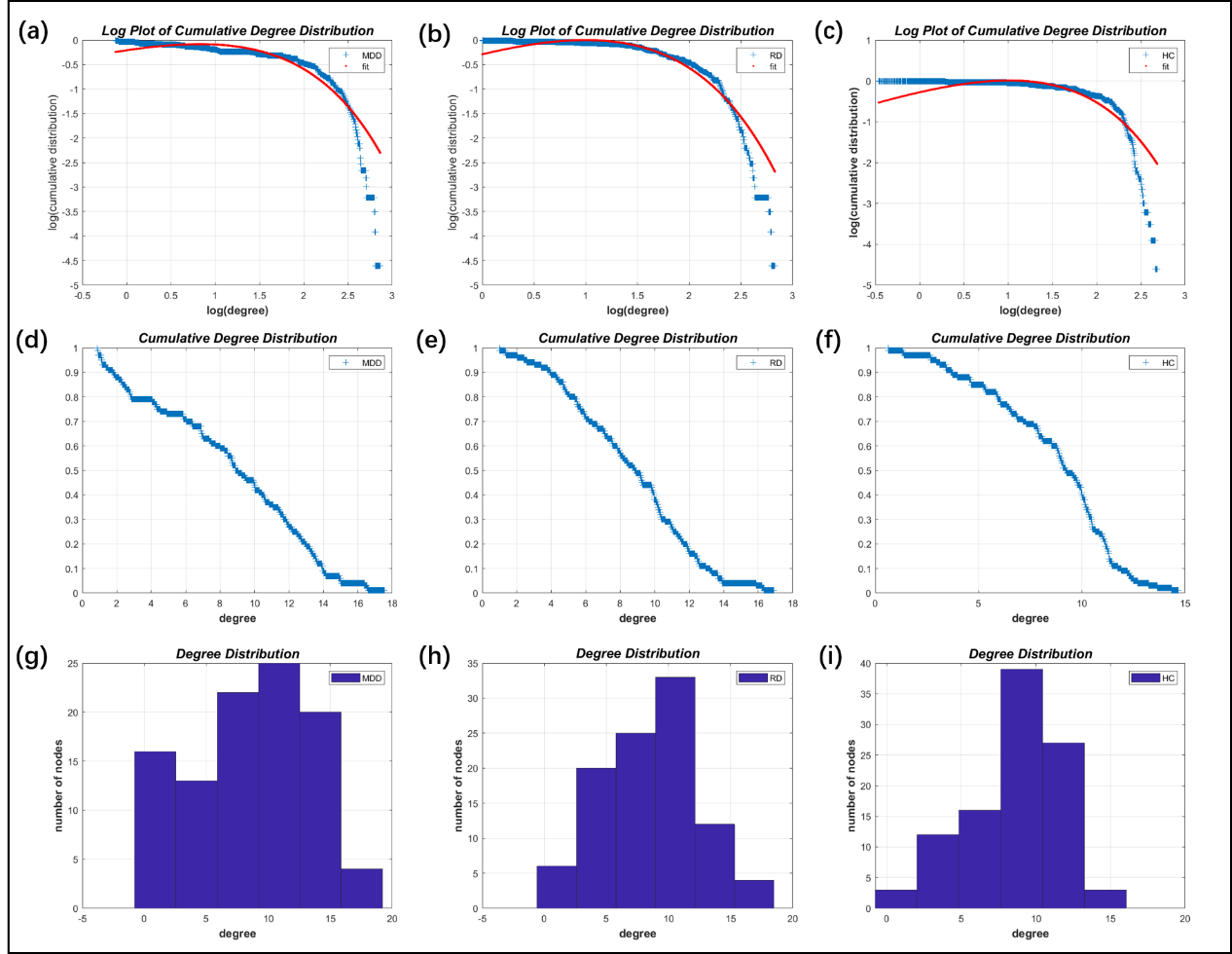


Fig. S3 Result of the degree distribution in (a, d, g) cMDD, (b, e, h) RD and (c, f, i) HC groups.


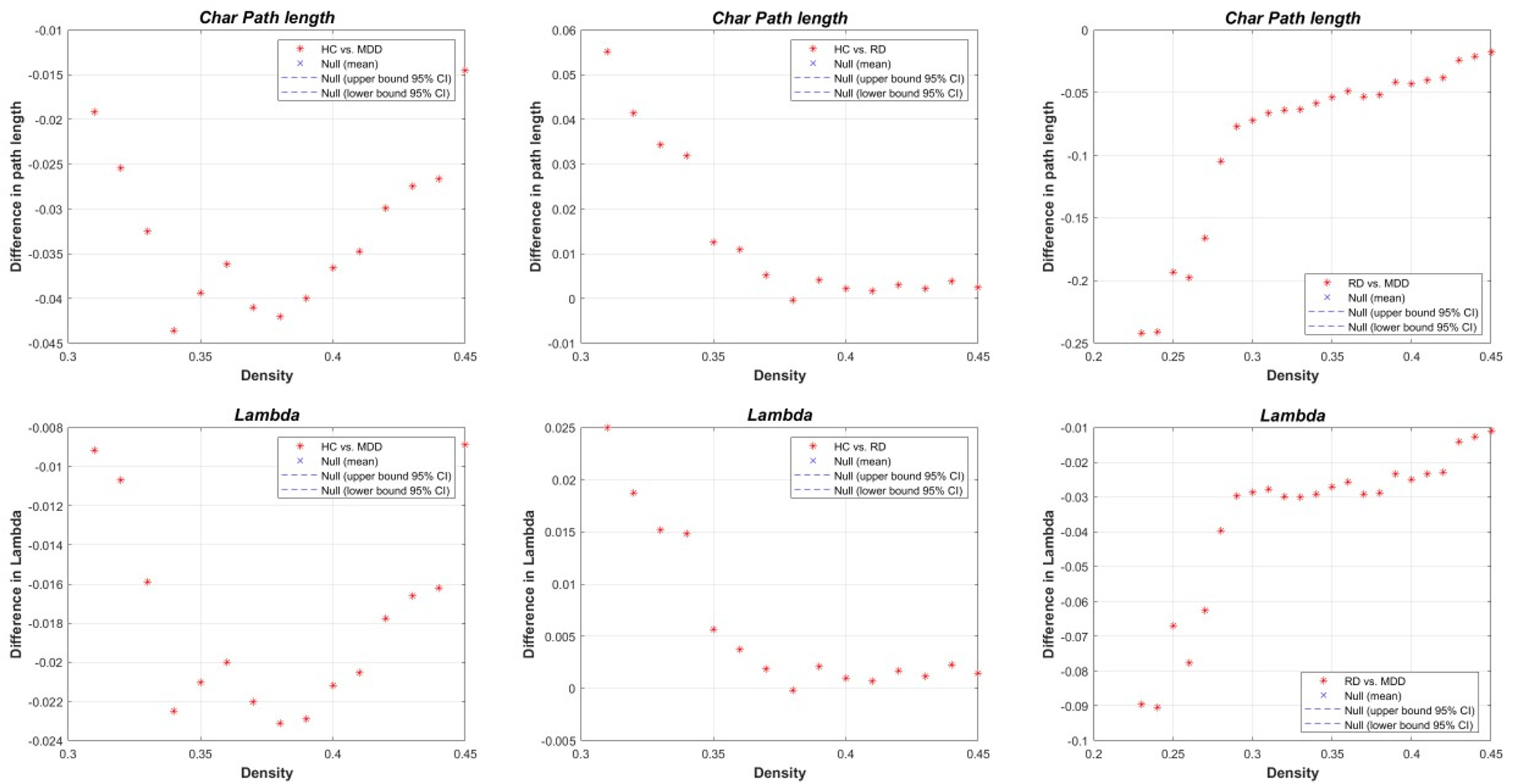


Fig. S4 Differences of Characteristic Path Length and Lamba (normalized path length) among the three Groups.


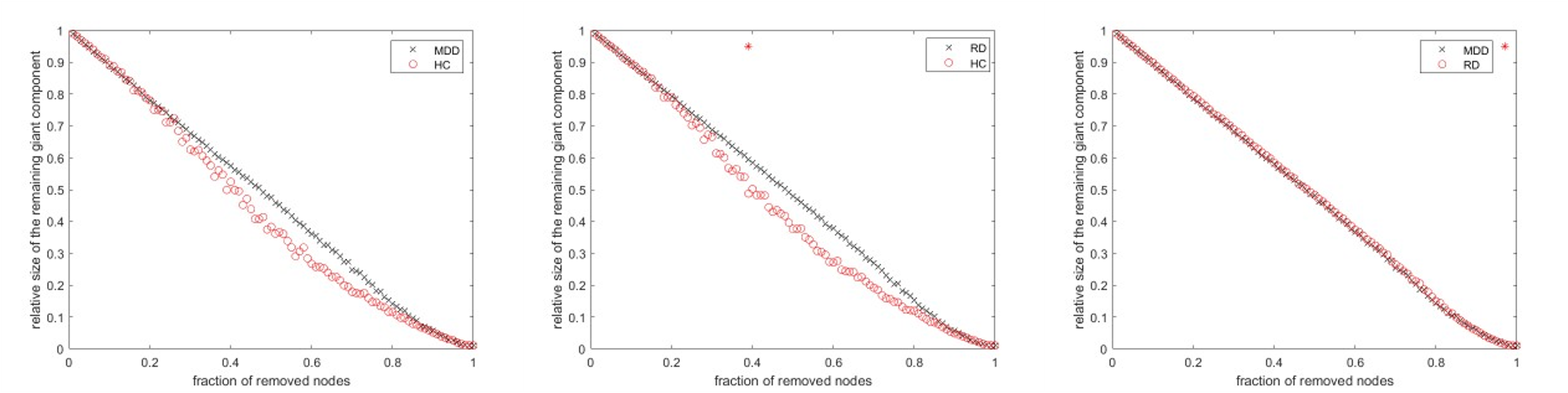


Fig. S5 Results of random attack among the three Groups.


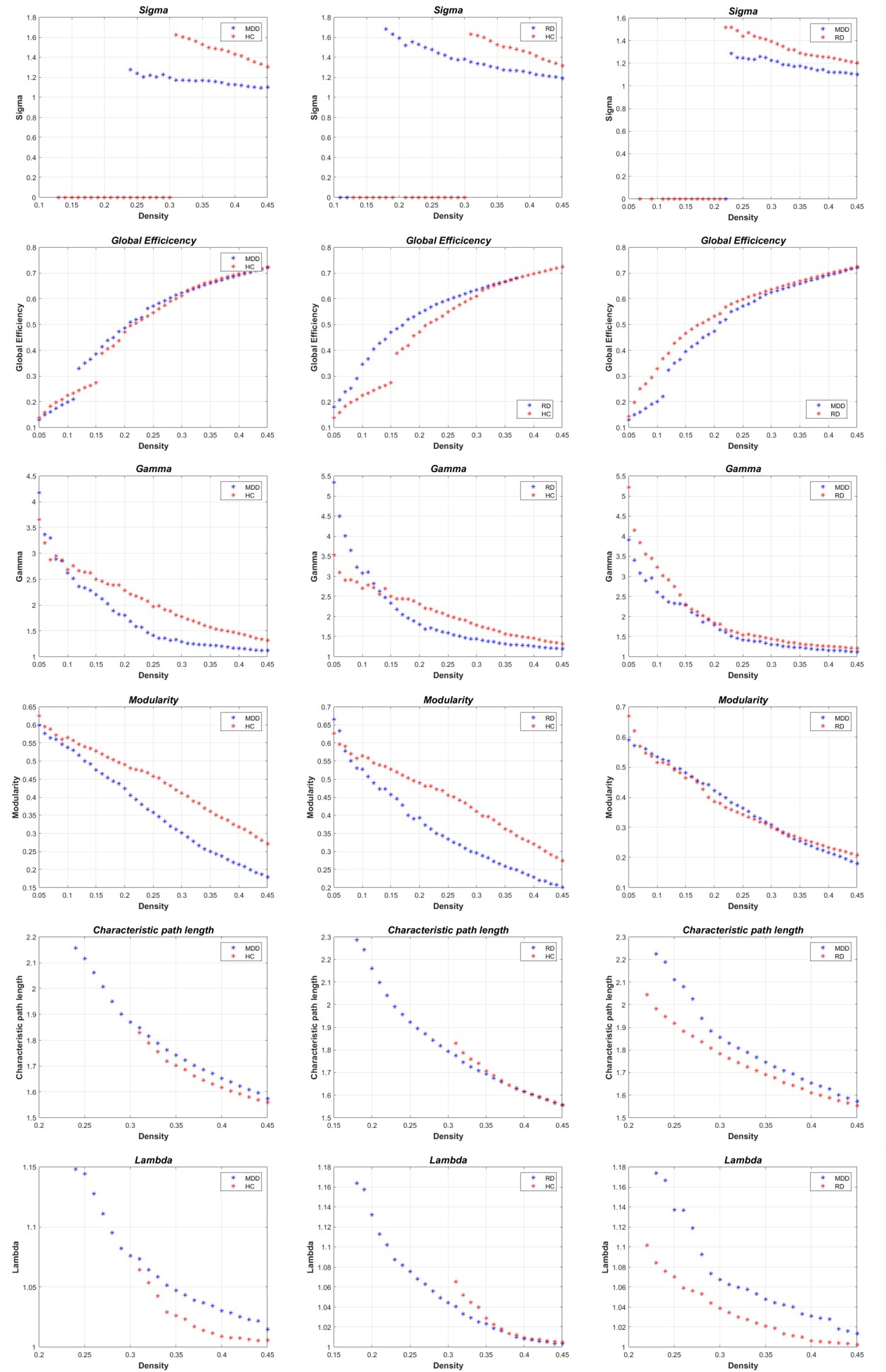


Fig. S6 Changes in Global Network Measures as a Function of Network Density (Sigma: small-world-ness; Gamma: normalized clustering coefficient; Lambda: normalized path length).
